# Care trajectories of women with advanced breast cancer at the end of life*

**DOI:** 10.1590/1980-220X-REEUSP-2025-0553en

**Published:** 2026-07-20

**Authors:** Danielle Copello Vaz, Ana Luisa Teixeira da Costa Durante, Livia Costa de Oliveira, Livia Gomes da Silva, Andrea dos Santos Garcia, Sonia Regina de Souza, Daniel Aragão Machado, Roberto Carlos Lyra da Silva, Suzi Maria Fernandes de Farias, Emanuel Pereira dos Santos, Davi da Silveira Barroso Alves, Carlos Roberto Lyra da Silva

**Affiliations:** 1 Instituto Nacional de Câncer, Hospital do Câncer IV, Ambulatório, Rio de Janeiro, RJ, Brazil.; 2 Instituto Nacional de Câncer, Hospital do Câncer IV, Internação hospitalar, Rio de Janeiro, RJ, Brazil.; 3 Instituto Nacional de Câncer, Hospital do Câncer III, Educação continuada, Rio de Janeiro, RJ, Brazil.; 4 Universidade Federal do Rio de Janeiro, Escola de Enfermagem Anna Nery, Enfermagem Médico-Cirúrgica, Rio de Janeiro, RJ, Brazil.; 5 Universidade Federal do Estado do Rio de Janeiro, Escola de Enfermagem Alfredo Pinto, Enfermagem Médico-Cirúrgica, Rio de Janeiro, RJ, Brazil.; 6 Universidade Federal do Estado do Rio de Janeiro, Escola de Enfermagem Alfredo Pinto, Enfermagem Fundamental, Rio de Janeiro, RJ, Brazil.; 7 Universidade Federal do Estado do Rio de Janeiro, Hospital Universitário Gaffree-Guinle, Anestesiologia, Rio de Janeiro, RJ, Brazil.; 8 Universidade Federal do Estado do Rio de Janeiro, Hospital Universitário Gaffree-Guinle, Pediatria, Rio de Janeiro, RJ, Brazil.; 9 Universidade Federal do Estado do Rio de Janeiro, Centro de Ciências Exatas e Tecnologia, Métodos Quantitativos, Rio de Janeiro, RJ, Brazil.

**Keywords:** Breast Neoplasms, Palliative Care, Patient Care, Indicators of Health Services, Death.

## Abstract

**Objective:**

To compare the care trajectories of women with advanced breast cancer in the last 30 days of life, analyzing clinical differences and healthcare service utilization between patients undergoing intravenous palliative chemotherapy (IPC) and those followed by specialized palliative care (SPC) teams.

**Method:**

A retrospective study based on the analysis of electronicmedical records of 370 women who died between 2019 and 2023, distributed into IPC (n = 176) and SPC (n = 194). Comparisons were performed using the chi-square, Fisher’s exact, or Mann–Whitney tests (p < 0.05).

**Results:**

Patients in SPC presented greater metastatic burden (4; IQR 3–5; p < 0.001), greater central nervous system involvement (39.69% vs. 16.48%; p < 0.001), and more multidisciplinary consultations (p < 0.001). They presented lower emergency care utilization (1; IQR 1–2; p < 0.001) and lower hospitalization in the last 24 hours of life (p < 0.001).

**Conclusion:**

Follow-up by SPC teams was associated with less aggressive care trajectories at the end of life, even among patients with greater clinical complexity.

## INTRODUCTION

Advanced breast cancer remains a global challenge, especially due to structural differences in access to timely diagnosis, disease-modifying treatments, and continuity of care. Recent evidence shows that, in 2022, breast cancer remained the most incident type among women, with approximately 2.3 million new cases and about 670 thousand deaths, while the mortality/incidence ratio ranges from 17% in highly developed countries to 56% in low-income countries. Projections indicate that, by 2050, a global increase of 68% in breast cancer deaths may occur, exceeding 160% in low- and middle-development countries, highlighting persistent disparities related to healthcare infrastructure, diagnostic capacity, and availability of therapies^(1)^. In this context, such inequalities are also reflected in the global provision of palliative care. An international ranking based on the regulatory framework of the World Health Organization evidenced profound differences among countries and regions regarding the implementation of palliative services, especially in low- and middle-income nations, where access remains limited and heterogeneous^(2)^.

Given disease progression, clinical and social needs become complex, requiring approaches focused on quality of life, symptom control, and shared decision-making. From this perspective, palliative care constitutes a multidimensional approach aimed at relieving suffering resulting from life-threatening conditions, integrating physical, psychosocial, spiritual, and existential dimensions continuously and in an articulated manner throughout the disease trajectory^(3)^. International guidelines emphasize that the early integration of palliative care should occur from the diagnosis of life-threatening diseases, in parallel with disease-modifying treatment, being complementary rather than substitutive to active therapies, including when systemic therapy is indicated^(4,5,6)^. This principle supports the systematic incorporation of palliative care into oncological treatment, especially in the context of advanced disease, favoring an integrated approach proportional to prognosis and centered on quality of life^(5,7)^. In this context, specialized palliative care (SPC) is mainly indicated in situations of greater clinical complexity, such as refractory symptoms or difficult-to-control suffering^(5,6)^.

The Brazilian National Palliative Care Policy advanced with the publication of Ordinance GM/MS 3,681/2024, which establishes guidelines and organizes services into levels of care, with quality-of-care monitoring within the Unified Health System^(8)^. By recognizing palliative care as a structuring element of the Healthcare Network, the regulation broadens the visibility of end-of-life care and establishes foundations for planning and articulation among levels of care, in addition to enabling assessment of care provided to individuals with life-threatening diseases. These directives align with the ethical and social commitments of the 2030 Agenda, which focuses on equity, the universal right to health, human dignity, and reduction of inequalities — dimensions that impact the care of individuals with advanced diseases^(9)^.

Despite advances consolidated in the international literature, especially regarding early integration of palliative care and reduction of aggressive care at the end of life^(5,7)^, comparative analyses are still needed to investigate how different care models are associated with patterns of care during the final period of life among women with advanced breast cancer, especially in the Brazilian context. Studies indicate that referral to SPC teams remains predominantly late, limiting opportunities for advance care planning and comfort-centered approaches^(10)^. Simultaneously, research demonstrates high utilization of hospital services and intensive interventions during the final months of life among oncology patients, reinforcing the persistence of potentially aggressive care patterns at the end of life^(11)^. In this scenario, it becomes relevant to compare how the persistence of active oncological treatment and follow-up by specialized palliative care teams relate to the configuration of end-of-life trajectories.

For conceptual purposes, a distinction is made between care trajectory and care model. Care trajectory refers to the concrete pathway experienced by patients within the healthcare system, including accessed services, transitions between levels of care, and interventions performed throughout illness and end of life. Care model, in turn, corresponds to the predominant organizational logic of care that guides clinical and institutional decisions. In the context of this study, the care model was operationalized as: (1) SPC, characterized by formal follow-up by a specialized team prioritizing comfort measures and symptom control; or (2) persistence of active oncological treatment with intravenous palliative chemotherapy (IPC), characterized by maintenance of disease-modifying interventions during the final period of life.

Thus, this study is justified by the need to analyze how different care models are associated with the configuration of care among women with advanced breast cancer at the end of life, contributing to the identification of care patterns and providing support for planning and organization of healthcare assistance. The hypothesis was that women followed by SPC teams would present lower frequency of interventions considered aggressive at the end of life when compared to those under persistence of active oncological treatment.

Given the above, this study aimed to compare the care trajectories of women with advanced breast cancer in the last 30 days of life, analyzing clinical differences and healthcare service utilization between patients undergoing IPC and those followed by SPC teams.

## METHOD

### Study Design

This is a retrospective, quantitative, observational, and analytical study based on secondary data extracted from electronic medical records. Two groups of women with advanced breast cancer who died between 2019 and 2023 were compared according to the predominant care model in the last 30 days of life.

### Study Setting

The study was conducted at a high-complexity public hospital, a national reference center in oncology, located in the state of Rio de Janeiro. The institution has a center dedicated to breast cancer treatment and an exclusive SPC unit, enabling comparison between different care trajectories.

### Population and Selection Criteria

Women with confirmed diagnosis of advanced breast cancer who died at the institution between January 2019 and December 2023 were included. Cases with a second primary tumor, diagnostic inconsistency (inadequate International Classification of Diseases or recording error), or COVID-19 in the last 30 days were excluded. The sample corresponded to all eligible cases during the analyzed period (n = 370).

The selection process involved identifying deaths in the institutional system, followed by application of the eligibility criteria and classification of patients according to the predominant care model in the 30 days preceding death. The comparison analyzed different care trajectories during the final period of life, considering healthcare organization as the structuring axis of the analysis. Thus, participants were grouped according to the predominant care model in the last 30 days of life.

The IPC group consisted of women who received IPC in the last 30 days of life and were followed in oncology services without inclusion in an SPC team. In this model, care remained structured under the logic of active oncological treatment, with predominant focus on maintaining antineoplastic therapies and reactive management of clinical complications.

The SPC group included women previously submitted to IPC throughout the care trajectory and who, in the last 30 days of life, were under exclusive follow-up by an SPC team, without receiving disease-modifying therapies during this period. The care model in this group was centered on symptom control and anticipation, shared decision-making, and interdisciplinary coordination of care.

Classification considered exclusively the predominant care model in the last 30 days of life, regardless of the timing of transition to SPC. All included patients received IPC at some point during the care trajectory. The cohorts differed regarding continuity or interruption of IPC in the final period of life.

At the studied institution, inclusion in SPC occurs after discontinuation of disease-modifying therapies, characterizing a sequential transition between active oncological treatment and SPC. Thus, patients are not simultaneously subjected to both care models. Furthermore, exclusive SPC follow-up does not include invasive advanced life-support measures, such as Intensive Care Unit admission, orotracheal intubation, or cardiopulmonary resuscitation. For this reason, such variables were not included in the comparative analysis between groups. This organizational characteristic should be considered when interpreting the findings, as it may influence patients’ clinical profile at the time of transition and the interventions performed during the final period of life.

### Data Collection and Analysis

Data collection was performed through retrospective analysis of institutional electronic medical records, the hospital management system, and complementary administrative databases. Data extraction was conducted by the principal researcher using the same standardized data collection form for both groups, specifically developed for this study based on the literature regarding indicators of therapeutic intensity and aggressiveness at the end of life in oncology^(11,12,13)^. The instrument was previously tested in a pilot sample to verify clarity and completeness of the fields. The form included sociodemographic, clinical, and care-related variables referring to the last 30 days of life.

The analyzed sociodemographic variables included age at institutional registration and at death, race, marital status, educational level, and religion. Clinical variables included metastatic burden, number and location of distant metastases, institutional follow-up time, and initial chemotherapy proposal. Institutional follow-up time corresponded to the interval between institutional registration and death.

Care indicators included chemotherapy administration near death, number of visits to the emergency care service (ECS), number of days of hospitalization, place of death, and late referral to SPC, variables described in the literature as markers of therapeutic intensity and potential aggressiveness of end-of-life care in oncology patients^(11,12,13)^.

Additionally, use of blood components and multidisciplinary consultations in the last 30 days of life were analyzed because they reflect hospital care intensity and the organizational pattern of care in the final period, enabling understanding of care trajectories between different care models. Analysis of multidisciplinary consultations was based on literature recognizing the interdisciplinary approach as a central component of palliative care quality and healthcare organization at the end of life^(4,14)^. The categories considered were physiotherapy, psychology, and social work, as these were the only professions with systematic records in electronic medical records in both assessed scenarios. These categories act non-continuously and on demand, allowing quantification of their presence in the different care models. Nursing, medicine, and nutrition were not included because they constitute continuous and mandatory presence during hospitalization, making their use as comparative variables unfeasible.

Clinical variables were included due to their prognostic relevance and potential influence on the definition of the care model and the intensity of interventions during the final period of life^(14,15,16)^. The temporal cutoff of the last 30 days was adopted according to consensus described in the international literature for assessing quality of end-of-life care^(11,12,13)^.

Data were organized into an electronic spreadsheet and analyzed using R software (version 4.3). Categorical variables were described through absolute and relative frequencies, whereas numerical variables were expressed as mean and standard deviation or median and interquartile range, according to data distribution. Comparisons between groups used the chi-square or Fisher’s exact tests for categorical variables and the Mann–Whitney U test for numerical variables, as they did not present normal distribution according to the Shapiro–Wilk test. A significance level of 5% was adopted.

### Ethical Aspects

The study was approved by the Research Ethics Committees of *Universidade Federal do Estado do Rio de Janeiro* (Opinion 6,845,555) and *Instituto Nacional de Câncer* (Opinion 6,950,714), with waiver of the Informed Consent Form due to the retrospective nature of the study.

## RESULTS

The sociodemographic characteristics of the 370 women with advanced breast cancer showed similarity between groups, without statistically significant differences. A higher proportion of mixed-race women was observed (46.49%), followed by White women (38.11%) and Black women (15.14%). Regarding educational level, incomplete elementary education or illiteracy predominated (36.22%), followed by secondary education (33.51%), while higher educational levels were less frequent (10.54%) and slightly more prevalent in the SPC group. In terms of marital status, the highest percentage corresponded to single women (39.73%), followed by married women or those in consensual unions (35.95%). Concerning religion, Catholic women predominated (45.68%), followed by Evangelical women (43.24%).

The distribution of the number of metastases differed between care models (Table 1), with statistical significance (p < 0.001). The IPC group presented higher frequency of three metastases (32.39%), whereas the SPC group presented higher proportions of four (27.32%) and five or more metastases (40.21%). Median metastases were 3 (IQR 2.75–4.00) among patients undergoing palliative chemotherapy and 4 (IQR 3–5) among those followed by SPC (p < 0.001). Regarding distant metastatic sites, greater occurrence of central nervous system metastases was observed among SPC patients (39.69% vs. 16.48%).

**Table 1:** Distribution of the number of sites and locations of distant metastases in patients with advanced breast cancer according to the type of care received – Rio de Janeiro, RJ, Brazil, 2025.

Variables	Total (n = 370^1^)	IPC (n = 176^1^)	SPC (n = 194^1^)	p-value^2^
**Number of metastases**				**< 0.001**
1	13 (3.51%)	10 (5.68%)	3 (1.55%)
2	51 (13.78%)	33 (18.75%)	18 (9.28%)
3	99 (26.76%)	57 (32.39%)	42 (21.65%)
4	89 (24.05%)	36 (20.45%)	53 (27.32%)
≥ 5	117 (31.62%)	39 (22.16%)	78 (40.20%)
Total metastases	4 (3–5)	3 (2.75–4.00)	4 (3–5)	**< 0.001**
**Sites of distant metastases**				
Liver	173 (46.76%)	84 (47.73%)	89 (45.88%)	0.722
Bone	232 (62.70%)	111 (63.07%)	121 (62.37%)	0.890
Pleural	95 (25.68%)	42 (23.86%)	53 (27.32%)	0.447
Lung	211 (57.03%)	105 (59.66%)	106 (54.64%)	0.330
Central nervous system	106 (28.65%)	29 (16.48%)	77 (39.69%)	**< 0.001**
Other^*^	60 (16.22%)	27 (15.34%)	33 (17.01%)	0.664

Legend: IPC – intravenous palliative chemotherapy; SPC – specialized palliative care.

*Others includes adrenal gland, bladder, stomach, bilateral breast, contralateral breast, mediastinum, bone marrow, ovary, parasternal region, soft tissues, pericardium, and peritoneum;

^1^Data expressed as absolute number (n) and percentage;

^2^Analyses performed using the chi-square test of independence. There was one case without record of the number of metastases.

In Table 2, it is observed that age at death differed between groups, being higher among patients followed by SPC (58 years; IQR 48–68) compared with those undergoing IPC (55 years; IQR 45–62) (p = 0.004). Institutional follow-up time was longer in the SPC group (53 months; IQR 30–102) compared with the IPC group (29 months; IQR 9–69) (p < 0.001). There was no difference regarding age at institutional registration (p = 0.373) or initial chemotherapy proposal (p = 0.312).

**Table 2:** Clinical and care indicators of patients with advanced breast cancer according to the type of care received* – Rio de Janeiro, RJ, Brazil, 2025.

Variables	Total (n = 370^1^)^*^	IPC (n = 176^1^)^*^	SPC (n = 194^1^)^*^	p-value^2^
Age at institutional registration^**^	50 (41–59)	50 (41–58)	51 (40–60)	0.373
Age at death^**^	56 (46–66)	55 (45–62)	58 (48–68)	0.004
Institutional follow-up time^***^	42 (17–87)	29 (9–69)	53 (30–102)	< 0.001
**Initial chemotherapy proposal**				0.312
Adjuvant	42 (11.35%)	17 (9.66%)	25 (12.89%)	
Neoadjuvant	178 (48.11%)	81 (46.02%)	97 (50.00%)	
Palliative	150 (40.54%)	78 (44.32%)	72 (37.11%)	
**Multidisciplinary consultations**				
Physical therapy	1 (0–3)	1 (0–3)	1 (0–2)	0.134
Psychology	0 (0–2)	0 (0–0)	2 (1–4)	< 0.001
Social work	1 (0–4)	0 (0–0)	4 (1.25–6)	< 0.001
**Received blood components**				
**Red blood cells**				< 0.001
Yes	38 (10.27%)	32 (18.18%)	6 (3.09%)	
No	332 (89.73%)	144 (81.82%)	188 (96.91%)	
**Platelets**				0.011
Yes	6 (1.62%)	6 (3.41%)	0 (0%)	
No	364 (98.38%)	170 (96.59%)	194 (100%)	
**Plasma**				0.606
Yes	3 (0.81%)	2 (1.14%)	1 (0.82%)	
No	367 (99.19%)	174 (98.86%)	193 (99.48%)	
**Number of ECS visits**	1 (1–2)	2 (1–3)	1 (1–2)	< 0.001
**Number of days hospitalized**	8 (4–13)	7 (4–12)	9 (3–14)	0.354
**Hospitalized in the last 72 hours**	256 (69.19%)	126 (71.59%)	130 (67.01%)	0.341
**Hospitalized in the last 48 hours**	285 (77.03%)	140 (79.55%)	145 (74.74%)	0.273
**Hospitalized in the last 24 hours**	331 (89.46%)	172 (97.73%)	159 (81.96%)	< 0.001
**Death site**				< 0.001
Hospitalization	331 (89.46%)	172 (97.73%)	159 (81.96%)	
Another hospital	19 (5.14%)	0 (0.00%)	19 (9.79%)	
On the way to the hospital	5 (1.35%)	1 (0.57%)	4 (2.06%)	
Home visit	10 (2.70%)	0 (0.0%)	10 (5.15%)	
Support house	2 (0.54%)	0 (0.00%)	2 (1.03%)	

Legend: IPC – intravenous palliative chemotherapy; SPC – specialized palliative care; ECS – emergency care service.

*The total number of analyzed medical records may vary due to missing information and does not necessarily correspond to the total sample (IPC: n = 176; SPC: n = 194);

variables marked with “**” are expressed in years and

those marked with “***” in months;

^1^Data expressed as median (25%–75%) or categorical values as absolute number (n) and percentage (%), as appropriate;

^2^Wilcoxon rank-sum test, chi-square test of independence, or Fisher’s exact test, as appropriate.

Concerning emergency service utilization, the number of ECS visits was higher in the IPC group (median of 2; IQR 1–3) compared with the SPC group (median of 1; IQR 1–2) (p < 0.001). The total number of hospitalization days in the last 30 days did not differ between groups (p = 0.354), with medians of nine days (IQR 3–14) in SPC and seven days (IQR 4–12) in IPC. In the analysis of hospitalization in the final days of life, a higher proportion of patients hospitalized in the last 24 hours was observed in the IPC group (97.73% vs. 81.96%; p < 0.001), without differences in the assessments of 72 and 48 hours (p>0.05).

In the last 30 days of life, differences between groups were observed regarding multidisciplinary consultations. Patients in SPC presented a higher number of psychology and social work consultations compared with IPC (p < 0.001 for both), whereas no difference was found in physiotherapy consultations (p = 0.134). Regarding the use of blood components, red blood cell transfusion was more frequent in IPC (18.2%) compared with SPC (3.1%) (p < 0.001), corresponding to 59 and 12 transfused units, respectively. Platelet use occurred exclusively in the IPC group (3.4%; p = 0.011). For plasma transfusion, no statistically significant difference was observed between groups (p = 0.606).

Most deaths occurred in the hospitals included in the study setting, with higher frequency among women undergoing IPC (97.73% vs. 81.96%; p < 0.001). Among patients followed by SPC, deaths occurred in other hospital institutions (9.79%), homes (5.15%), support houses (1.03%), and during transportation (2.06%). In the IPC group, there were no home or support house deaths; one death occurred during transportation (0.57%); and three medical records lacked information regarding place of death.

## DISCUSSION

The analyzed sociodemographic characteristics did not exert significant influence on the care trajectory at the end of life, since the groups presented similar distributions regarding race, educational level, marital status, and religion. Given this homogeneity, the differences observed between the assessed care models do not appear to be related to social inequalities, but possibly to the way specialized care was incorporated into oncological treatment. This pattern suggests that clinical criteria, disease progression, and institutional practices regarding healthcare organization may have played a relevant role in shaping care trajectories. The distribution of the initial treatment proposal demonstrates that the institution predominantly receives women in locally advanced or metastatic stages, with predominance of neoadjuvant or directly palliative chemotherapy and reduced adjuvant indication, a pattern compatible with the still predominant late diagnosis in Brazil^(17,18)^.

In this scenario, the longer institutional follow-up time observed among patients referred to SPC (interval between institutional registration and death) may indicate that, after an initial period of disease control, progression and gradual reduction of disease-modifying treatment options occurred, culminating in referral to SPC, generally when clinical irreversibility was already established. This organizational arrangement suggests that referral to SPC occurs predominantly late, after exhaustion of disease-modifying therapies, in contrast with recommendations advocating early and parallel integration of palliative care into active oncological treatment at different levels of care, including initial management by the care team itself and referral to specialized teams in cases of greater clinical or symptomatic complexity^(5,6)^.

In IPC may reflect the more aggressive biology of certain tumors, a characteristic particularly described among younger women, who present greater frequency of unfavorable subtypes and need for more intense systemic treatment throughout the disease trajectory^(19)^. Evidence from a large prospective cohort demonstrates that, in the context of advanced disease, the effectiveness of palliative systemic therapies depends on tumor biology, with wide variation in survival even after multiple lines of treatment^(20)^. In this context, the findings of the present study, which identified lower age at death among those maintained on palliative chemotherapy, are compatible with this clinical pattern and suggest the complexity of therapeutic management in more aggressive tumors. However, persistence of antineoplastic treatments, even in the face of clinical irreversibility, remains consistent with the phenomenon of therapeutic inertia, described in the literature as a factor associated with delayed integration of palliative care and risk of disproportionate interventions at the end of life^(21)^.

In light of these findings, the set of interventions observed at the end of life may be interpreted through the concept of therapeutic obstinacy or futility, widely discussed in the literature. This concept refers to the maintenance of biomedical interventions with low probability of proportional benefit in the face of clinical irreversibility, frequently associated with persistence of disease-modifying treatments, use of supportive procedures with limited benefit, and absence of advance care planning^(11,12)^. Recent studies indicate that fragmentation of care and late integration of SPC favor trajectories of greater therapeutic intensity, including chemotherapy near death, repeated hospitalizations, and potentially disproportionate supportive interventions^(11,12,13,22)^.

The clinical severity observed at the time of SPC admission also reinforces this care dynamic. In this group, the greater metastatic burden and high frequency of central nervous system involvement indicate that palliative care assumes follow-up only when the disease already presents complex and potentially disabling complications, limiting the impact of preventive strategies and advance care planning. The late incorporation of SPC suggests a predominantly reactive care model, centered on therapeutic failure rather than progressive and parallel integration with oncological treatment. This pattern aligns with the international literature, which points to structural, cultural, and organizational barriers to early integration of palliative care, even in contexts where its provision is recommended from the diagnosis of life- threatening diseases^(4,5,6)^.

Despite the more advanced clinical condition, patients followed by SPC did not present greater consumption of hospital resources. Lower use of emergency services, lower transfusional demand, and lower frequency of hospitalization in the specialized hospital during the last 24 hours of life were verified. This finding suggests that reduction in care aggressiveness at the end of life does not result solely from clinical severity, but from the way care is organized, particularly regarding symptom anticipation and decision-making. Maintenance of chemotherapy near death, a practice widely documented as associated with worsening quality of life and increased intensity of acute interventions, has limited clinical benefit and may reduce opportunities for therapeutic planning centered on comfort^(22,23)^.

Another aspect reinforcing the pattern of greater therapeutic intensity among patients undergoing palliative chemotherapy was the use of blood components during the final weeks of life. Greater transfusional demand was observed in this group, with administration of red blood cells and platelets up to the final days before death. Although transfusions may provide transient symptomatic benefit, studies indicate that such effects are brief and minimally expressive in terminal-phase patients^(24)^. In a retrospective study involving 179 patients with advanced cancer followed in palliative care, only 36% presented clinical benefit, generally limited to 15 days after the procedure, with median survival of 41 days, reinforcing the limited benefit of this intervention in individuals with poor functional status^(25)^.

Although no statistically significant difference was observed in the total number of hospitalization days between groups, the slightly higher median observed among patients followed by SPC (nine vs. seven days) should be interpreted cautiously, since the absence of statistical significance does not allow affirmation of a direct association between the care model and hospitalization duration. Still, this difference may reflect the more complex clinical profile of this group, characterized by greater metastatic burden and central nervous system involvement, frequently associated with neurological symptoms, rapid functional decline, and reduced therapeutic options^(26)^. Additionally, multiple metastatic sites and poorer general condition are among the factors associated with worse survival in advanced breast cancer^(27)^. However, the greater need for care did not translate into greater utilization of acute interventions, suggesting the modulating role of SPC in crisis prevention and anticipatory symptom management, avoiding reactive hospitalizations and favoring therapeutic decisions aligned with care goals.

The mechanisms associated with lower resource utilization and lower care aggressiveness observed among patients followed by SPC may be understood through three dimensions described in the literature on early integration of palliative care. The first refers to anticipatory symptom management and continuous clinical surveillance, capable of reducing acute decompensations and preventing crises that frequently motivate emergency visits^(4,5)^. The second involves shared decision-making and advance care planning, which allow alignment of expectations, avoidance of futile interventions, and reorientation of therapeutic priorities in the face of clinical irreversibility^(4,6)^. The third relates to interdisciplinary coordination of the care trajectory, integrating physical, emotional, social, and spiritual dimension^(5,7,28)^.

In this study, patients followed by SPC received a greater number of psychology and social work consultations during the final weeks of life, evidencing the implementation of a multidisciplinary approach directed toward management of emotional and social suffering, recognized as an essential component of oncology care quality^(28)^. These mechanisms favor therapeutic choices more aligned with patients’ values, reducing precipitated decisions during acute crises and modulating the pattern of hospitalization at the end of life^(4,6)^. Recent evidence reinforces the relevance of this integrated model, demonstrating that fragmentation of care and neglect of emotional, social, spiritual, existential, and relational dimensions constitute sources of avoidable suffering and favor reactive patterns of care^(28)^. Overcoming such gaps requires multidisciplinary teams capable of offering psychological, social, and spiritual support throughout the disease trajectory, promoting decisions consistent with values, preventing disproportionate interventions, and modulating resource utilization at the end of life^(3)^. These elements dialogue with the findings of this study, suggesting that the active presence of SPC teams contributes to less aggressive, more continuous care aligned with patients’ needs and preferences.

Additionally, an increase in the proportion of hospitalized patients was observed as death approached, corresponding to 69.19% in the last 72 hours, 77.03% in the last 48 hours, and 89.46% in the final 24 hours. Although terminal clinical decline is frequently accompanied by the need for hospitalization to manage intense symptoms such as pain, dyspnea, agitation, and terminal delirium^(4)^, a significant difference between groups was observed only in the last 24 hours of life, with a higher proportion of hospitalization among patients undergoing palliative chemotherapy (97.73%) compared with those followed by SPC (81.96%; p < 0.001). This pattern may reflect maintenance of disease-modifying therapeutic strategies until phases very close to death, increasing dependence on hospital support and the probability of intensive interventions during the final hours of life. In contexts where advance care planning is limited or occurs late, persistence of these strategies may favor reactive hospitalizations and greater therapeutic intensity during the final period of life.

Among patients followed by SPC, a higher proportion of deaths outside the reference hospital was identified, including other hospitals (9.79%) and home (5.15%). Although such findings do not allow inference of a direct causal relationship between the care model and place of death, they suggest that continuous clinical support, advance planning, and territorial articulation may reduce the need for hospitalization in high- complexity hospitals, redistributing care according to clinical needs and network availability. In the Brazilian context, a national study demonstrates that, although most oncology patients express the desire to die at home, this outcome is frequently hindered by family insecurity, absence of specialized support, and communication failures in the care process^(29)^.

The SPC model analyzed in this study is characterized by articulation between outpatient care, home follow-up, and telemonitoring, integrated with Primary Health Care (PHC) and lower-complexity hospital units, favoring continuity of care in locations close to home when clinically appropriate. In these contexts, hospitalization is not necessarily avoided but may be relocated more fluidly within the territory, potentially reducing exclusive dependence on the specialized hospital and allowing care in units more appropriate to the clinical situation and family conditions. This organization aligns with recent guidelines for palliative care organization within the Brazilian healthcare system. Ordinance GM/MS 3,681/2024 emphasizes territorialization and intersectoral coordination of palliative care, integrating specialized services, PHC, and communication technologies, aiming to ensure continuity of care, reduce unnecessary displacement, and support shared decisions regarding place of death. By expanding possibilities for care outside the high-complexity hospital, this healthcare organization reinforces the relevance of articulated networks and person-centered strategies, favoring care pathways compatible with clinical needs, preferences, and dignity in the dying process^(8)^. This perspective also aligns with the 2030 Agenda, particularly Sustainable Development Goal 3 and its target 3.8, which promotes universal health coverage, in which palliative care is recognized as an essential component^(9)^.

In this context, the results of this study also allow reflection on possible implications for nursing practice in the coordination and continuity of care throughout the disease trajectory. Nursing professionals, because they remain in more frequent contact with patients and family members, play a strategic role in continuous clinical surveillance, early identification of symptoms, and facilitation of communication among the team, patients, and caregivers. This performance favors early recognition of palliative needs, contributes to care planning, and may support therapeutic decisions more aligned with care goals and patients’ clinical conditions. Recent evidence indicates that nurses with training in oncology and palliative care may contribute to integrating these approaches into oncology practice, expanding healthcare teams’ capacity to identify palliative needs early and support symptom management and communication with patients and family members throughout the disease trajectory^(30)^.

The results of this study reinforce that the differences observed between groups seem to derive mainly from the way care is organized throughout the disease trajectory, evidencing the influence of the care model on the intensity of interventions at the end of life. Recent international studies indicate that oncology systems that structurally articulate palliative care, even when this integration occurs late, tend to favor greater coherence between clinical interventions, patients’ needs, and care goals, with reduction of disproportionate procedures and better therapeutic alignment^(7)^. In light of these references, the findings of this investigation contribute to demonstrating that the active presence of SPC teams, even when introduced only in advanced phases of disease, plays a relevant role in modulating resource utilization, preventing futile interventions, and promoting more continuous, safer, and person-centered care at the end of life.

This study presents limitations inherent to the retrospective design and the exclusive use of secondary data, dependent on the quality and completeness of electronic records. The compared groups were not randomly constituted, which may have introduced selection bias, especially because referral to SPC tends to occur in more advanced stages of disease. The analysis focused on a single public reference institution, which may restrict generalization of the results to settings with lower availability of supplies, multidisciplinary teams, or territorial support. Psychosocial variables, patients’ preferences, or measures of perceived quality of care were not assessed, which could deepen understanding of the end-of-life trajectory. Furthermore, multivariate models were not applied, since the study focus was descriptive and comparative, without intention to produce causal inferences. Despite these limitations, the findings provide evidence still scarcely explored in the Brazilian context by demonstrating differences between care models at the end of life, contributing to improvement of SPC and to planning of public policies in oncology.

## CONCLUSION

The findings of this study indicate that the predominant care model in the last 30 days of life was associated with different patterns of healthcare service utilization among women with advanced breast cancer. Even with similar sociodemographic profiles, patients followed by SPC presented lower utilization of emergency services, lower use of blood components, and lower frequency of hospitalization in the last 24 hours of life in specialized hospitals, despite presenting greater metastatic burden, including greater central nervous system involvement.

Although causal relationships cannot be established, the results suggest that the way care is organized may influence the therapeutic trajectory at the end of life. Strategies such as multidisciplinary support, structured communication, advance care planning, and articulation among services may contribute to greater alignment between clinical decisions and care goals at the end of life. In this scenario, it is possible to reflect on the potential implications for nursing practice in care coordination and care navigation throughout the disease trajectory, contributing to therapeutic decisions more aligned with patients’ needs and priorities at the end of life.

Future investigations, especially those using prospective designs, may contribute to analyzing how clinical factors and characteristics of care models relate to end-of-life outcomes, expanding understanding regarding strategies that favor proportional, person-centered care aligned with the needs of patients with advanced cancer.

## DATA AVAILABILITY

The entire dataset supporting the results of this study has been published in the article itself.
