# Supplementary material for: Care trajectories of women with advanced breast cancer at the end of life
Source: Rev Esc Enferm USP. 2026 Jul 20;60:e20250553. doi: 10.1590/1980-220X-REEUSP-2025-0553en (PMC13390997; doi:10.1590/1980-220X-REEUSP-2025-0553en)
Supplement: Translation

# Translation: Trajetórias assistenciais de mulheres com câncer de mama avançado no final da vida*

**DOI:** 10.1590/1980-220X-REEUSP-2025-0553pt

**Authors:** Danielle Copello Vaz, Ana Luisa Teixeira da Costa Durante, Livia Costa de Oliveira, Livia Gomes da Silva, Andrea dos Santos Garcia, Sonia Regina de Souza, Daniel Aragão Machado, Roberto Carlos Lyra da Silva, Suzi Maria Fernandes de Farias, Emanuel Pereira dos Santos, Davi da Silveira Barroso Alves, Carlos Roberto Lyra da Silva

**Affiliations:** 1 Instituto Nacional de Câncer, Hospital do Câncer IV, Ambulatório, Rio de Janeiro, RJ, Brasil.; 2 Instituto Nacional de Câncer, Hospital do Câncer IV, Internação hospitalar, Rio de Janeiro, RJ, Brasil.; 3 Instituto Nacional de Câncer, Hospital do Câncer III, Educação continuada, Rio de Janeiro, RJ, Brasil.; 4 Universidade Federal do Rio de Janeiro, Escola de Enfermagem Anna Nery, Enfermagem Médico-Cirúrgica, Rio de Janeiro, RJ, Brasil.; 5 Universidade Federal do Estado do Rio de Janeiro, Escola de Enfermagem Alfredo Pinto, Enfermagem Médico-Cirúrgica, Rio de Janeiro, RJ, Brasil.; 6 Universidade Federal do Estado do Rio de Janeiro, Escola de Enfermagem Alfredo Pinto, Enfermagem Fundamental, Rio de Janeiro, RJ, Brasil.; 7 Universidade Federal do Estado do Rio de Janeiro, Hospital Universitário Gaffree-Guinle, Anestesiologia, Rio de Janeiro, RJ, Brasil.; 8 Universidade Federal do Estado do Rio de Janeiro, Hospital Universitário Gaffree-Guinle, Pediatria, Rio de Janeiro, RJ, Brasil.; 9 Universidade Federal do Estado do Rio de Janeiro, Centro de Ciências Exatas e Tecnologia, Métodos Quantitativos, Rio de Janeiro, RJ, Brasil.

**Keywords:** Neoplasias da Mama, Cuidados Paliativos, Assistência ao Paciente, Indicadores de Serviços, Terminalidade da Vida.

## Abstract

**Objetivo:**

Comparar as trajetórias assistenciais de mulheres com câncer de mama avançado nos últimos 30 dias de vida, analisando diferenças clínicas e de utilização de serviços de saúde entre pacientes submetidas à quimioterapia paliativa endovenosa (QPE) e aquelas acompanhadas por equipes de cuidados paliativos especializados (CPE).

**Método:**

Estudo retrospectivo, baseado na análise de prontuários eletrônicos de 370 mulheres que evoluíram a óbito entre 2019 e 2023, distribuídas em QPE (n = 176) e CPE (n = 194). As comparações utilizaram os testes qui-quadrado, exato de Fisher ou Mann–Whitney (p < 0,05).

**Resultados:**

Pacientes em CPE apresentaram maior carga metastática (4; IIQ 3–5; p < 0,001), maior acometimento do sistema nervoso central (39,69% vs. 16,48%; p < 0,001) e mais atendimentos multiprofissionais (p < 0,001). Apresentaram menor uso de pronto atendimento (1; IIQ 1–2; p < 0,001) e menor internação nas últimas 24 horas de vida (p < 0,001).

**Conclusão:**

O acompanhamento por equipes de CPE esteve associado a trajetórias assistenciais menos agressivas no final da vida, mesmo em pacientes com maior complexidade clínica.

## INTRODUÇÃO

O câncer de mama avançado permanece um desafio global, sobretudo devido às diferenças estruturais no acesso ao diagnóstico oportuno, aos tratamentos modificadores da doença e à continuidade do cuidado. Evidências recentes mostram que, em 2022, o câncer de mama manteve-se como o tipo mais incidente entre as mulheres, com aproximadamente 2,3 milhões de novos casos e cerca de 670 mil mortes, sendo que a razão mortalidade/incidência varia de 17% em países com alto desenvolvimento a 56% em países de baixa renda. Projeções indicam que, até 2050, poderá ocorrer um aumento global de 68% nas mortes por câncer de mama, ultrapassando 160% em países de médio e baixo desenvolvimento, o que evidencia disparidades persistentes relacionadas à infraestrutura assistencial, à capacidade diagnóstica e à oferta de terapias^(1)^. Nesse contexto, tais desigualdades também se refletem na oferta global de cuidados paliativos. Um *ranking* internacional baseado no marco regulatório da Organização Mundial da Saúde evidenciou profundas diferenças entre países e regiões na implementação de serviços paliativos, especialmente em nações de baixa e média renda, onde o acesso permanece limitado e heterogêneo^(2)^.

Diante da progressão da doença, as necessidades clínicas e sociais tornam-se complexas, exigindo abordagens voltadas à qualidade de vida, ao controle de sintomas e à tomada de decisão compartilhada. Nessa perspectiva, os cuidados paliativos configuram uma abordagem multidimensional orientada ao alívio do sofrimento decorrente de condições que ameaçam a vida, integrando dimensões físicas, psicossociais, espirituais e existenciais, de maneira contínua e articulada, ao longo da trajetória da doença^(3)^. Diretrizes internacionais destacam que a integração precoce dos cuidados paliativos deve ocorrer desde o diagnóstico de doenças ameaçadoras da vida, de forma paralela ao tratamento modificador da doença, sendo complementar e não substitutiva das terapias ativas, inclusive quando há indicação de terapia sistêmica^(4,5,6)^. Esse princípio sustenta a incorporação sistemática dos cuidados paliativos ao tratamento oncológico, especialmente no contexto da doença avançada, favorecendo uma abordagem integrada, proporcional ao prognóstico e centrada na qualidade de vida^(5,7)^. Nesse contexto, os cuidados paliativos especializados (CPE) são indicados principalmente em situações de maior complexidade clínica, como sintomas refratários ou sofrimento de difícil controle^(5,6)^.

No Brasil, a Política Nacional de Cuidados Paliativos avançou com a publicação da Portaria GM/MS n^o^ 3.681/2024, que estabelece diretrizes e organiza os serviços em níveis assistenciais, com monitoramento da qualidade do cuidado no Sistema Único de Saúde^(8)^. Ao reconhecer os cuidados paliativos como elemento estruturante da Rede de Atenção à Saúde, a norma amplia a visibilidade dos cuidados no fim da vida e estabelece bases para o planejamento e a articulação entre os níveis de assistência, além de possibilitar a avaliação da assistência às pessoas com doenças que colocam a vida em risco. Esses direcionamentos dialogam com os compromissos éticos e sociais da Agenda 2030, que tem como foco a equidade, o direito universal à saúde, a dignidade humana e a redução de desigualdades — dimensões que impactam o cuidado de pessoas com doenças avançadas^(9)^.

Apesar dos avanços consolidados na literatura internacional, especialmente no que se refere à integração precoce dos cuidados paliativos e à redução da agressividade assistencial no final da vida^(5,7)^, ainda são necessárias análises comparativas que investiguem como diferentes modelos assistenciais se associam a padrões de cuidado no período final da vida de mulheres com câncer de mama avançado, especialmente no contexto brasileiro. Estudos indicam que o encaminhamento para equipes de CPE permanece predominantemente tardio, restringindo oportunidades de planejamento antecipado e de abordagem centrada no conforto^(10)^. Paralelamente, pesquisas evidenciam elevada utilização de serviços hospitalares e intervenções intensivas nos últimos meses de vida de pacientes oncológicos, reforçando a persistência de padrões assistenciais potencialmente agressivos no final da vida^(11)^. Nesse cenário, torna-se relevante comparar como a persistência de tratamento oncológico ativo e o acompanhamento por equipe especializada em cuidados paliativos se relacionam com a configuração das trajetórias no final da vida.

Para fins conceituais, distingue-se trajetória assistencial de modelo assistencial. Trajetória assistencial refere-se ao percurso concreto vivenciado pela paciente no sistema de saúde, incluindo serviços acessados, transições entre níveis de atenção e intervenções realizadas ao longo do adoecimento e do final da vida. Modelo assistencial, por sua vez, corresponde à lógica organizadora predominante do cuidado que orienta decisões clínicas e institucionais. No contexto deste estudo, o modelo assistencial foi operacionalizado como: (1) CPE, caracterizados pelo acompanhamento formal por equipe especializada com priorização de medidas de conforto e controle sintomático; ou (2) persistência de tratamento oncológico ativo com quimioterapia paliativa endovenosa (QPE), caracterizada pela manutenção de intervenções modificadoras da doença no período final da vida.

Assim, este estudo se justifica pela necessidade de analisar como diferentes modelos assistenciais se associam à configuração do cuidado de mulheres com câncer de mama avançado no final da vida, contribuindo para a identificação de padrões assistenciais e oferecendo subsídios para o planejamento e a organização da assistência. Parte-se da hipótese de que mulheres acompanhadas por CPE apresentam menor frequência de intervenções consideradas agressivas no final da vida quando comparadas àquelas sob persistência de tratamento oncológico ativo.

Diante do exposto, este estudo teve como objetivo comparar as trajetórias assistenciais de mulheres com câncer de mama avançado nos últimos 30 dias de vida, analisando diferenças clínicas e de utilização de serviços de saúde entre pacientes submetidas à QPE e aquelas acompanhadas por equipes de CPE.

## MÉTODO

### Tipo de Estudo

Trata-se de estudo retrospectivo, quantitativo, observacional e analítico, baseado em dados secundários extraídos de prontuários eletrônicos. Foram comparados dois grupos de mulheres com câncer de mama avançado que evoluíram a óbito entre 2019 e 2023, segundo o modelo assistencial predominante nos últimos 30 dias de vida.

### Local do Estudo

A pesquisa foi realizada em um hospital público de alta complexidade, referência nacional em oncologia, localizado no estado do Rio de Janeiro. A instituição dispõe de centro dedicado ao tratamento do câncer de mama e de unidade exclusiva de CPE, possibilitando a comparação entre diferentes trajetórias assistenciais.

### População e Critérios de Seleção

Foram incluídas mulheres com diagnóstico confirmado de câncer de mama avançado que evoluíram a óbito na instituição entre janeiro de 2019 e dezembro de 2023. Foram excluídos os casos com segundo tumor primário, inconsistência diagnóstica (Classificação Internacional de Doenças inadequada ou erro de registro) ou COVID-19 nos últimos 30 dias. A amostra correspondeu à totalidade dos casos elegíveis no período analisado (n = 370).

O processo de seleção envolveu a identificação dos óbitos no sistema institucional, seguida da aplicação dos critérios de elegibilidade e da classificação das pacientes quanto ao modelo assistencial predominante nos 30 dias que antecederam o óbito. A comparação analisou diferentes trajetórias de cuidado no período final da vida, considerando a organização assistencial como eixo estruturante da análise. Para isso, as participantes foram agrupadas conforme o modelo assistencial predominante nos últimos 30 dias de vida.

O grupo QPE foi composto por mulheres que receberam QPE nos últimos 30 dias de vida, acompanhadas em serviços oncológicos sem inserção em equipe de CPE. Nesse modelo, o cuidado permaneceu estruturado sob a lógica do tratamento oncológico ativo, com foco predominante na manutenção de terapias antineoplásicas e no manejo reativo de intercorrências clínicas.

O grupo CPE incluiu mulheres previamente submetidas à QPE ao longo da trajetória assistencial e que, nos últimos 30 dias de vida, encontravam-se em acompanhamento exclusivo por equipe de CPE, sem recebimento de terapias modificadoras da doença nesse período. O modelo assistencial nesse grupo foi centrado no controle e na antecipação de sintomas, na tomada de decisão compartilhada e na coordenação interdisciplinar do cuidado.

A classificação considerou exclusivamente o modelo assistencial predominante nos últimos 30 dias de vida, independentemente do momento da transição para o CPE. Todas as pacientes incluídas receberam QPE em algum momento da trajetória assistencial. As coortes distinguiram-se quanto à continuidade ou interrupção da QPE no período final da vida.

Na instituição estudada, a inserção no CPE ocorre após a suspensão das terapias modificadoras da doença, caracterizando transição sequencial entre tratamento oncológico ativo e CPE. Assim, as pacientes não são submetidas simultaneamente aos dois modelos assistenciais. Além disso, o acompanhamento exclusivo por CPE não inclui medidas invasivas de suporte avançado de vida, como admissão em Unidade de Terapia Intensiva, intubação orotraqueal ou reanimação cardiopulmonar. Por essa razão, tais variáveis não foram incluídas na análise comparativa entre os grupos. Essa característica organizacional deve ser considerada na interpretação dos achados, pois pode influenciar o perfil clínico das pacientes no momento da transição e as intervenções realizadas no período final de vida.

### Coleta e Análise dos Dados

A coleta de dados foi realizada por meio de análise retrospectiva dos prontuários eletrônicos institucionais, do sistema de gestão hospitalar e de bancos administrativos complementares. A extração dos dados foi realizada pela pesquisadora principal, utilizando a mesma ficha padronizada de coleta de dados para ambos os grupos, elaborada especificamente para o estudo com base na literatura sobre indicadores de intensidade terapêutica e agressividade no final da vida em oncologia^(11,12,13)^. O instrumento foi previamente testado em amostra piloto para verificar a clareza e completude dos campos. A ficha contemplou variáveis sociodemográficas, clínicas e assistenciais referentes aos últimos 30 dias de vida.

Foram analisadas variáveis sociodemográficas (idade na matrícula e no óbito, raça, estado civil, escolaridade e religião), variáveis clínicas (carga metastática, número e localização das metástases à distância, tempo de seguimento institucional e proposta inicial da quimioterapia) e indicadores assistenciais referentes ao final da vida. O tempo de seguimento institucional corresponde ao intervalo entre a matrícula institucional da paciente e o óbito.

Os indicadores assistenciais incluíram administração de quimioterapia próxima ao óbito, número de visitas ao serviço de pronto atendimento (SPA), número de dias de internação hospitalar, local do óbito e encaminhamento tardio aos CPE, variáveis descritas na literatura como marcadores de intensidade terapêutica e potencial agressividade do cuidado no final da vida em pacientes oncológicos^(11,12,13)^.

Adicionalmente, foram analisados o uso de hemocomponentes e o atendimento multiprofissional nos últimos 30 dias de vida por refletirem a intensidade assistencial hospitalar e o padrão organizacional dos cuidados no período final, permitindo compreender as trajetórias assistenciais entre os diferentes modelos. A análise do atendimento multiprofissional foi fundamentada na literatura que reconhece a abordagem interdisciplinar como componente central da qualidade dos cuidados paliativos e da organização assistencial no fim da vida^(4,14)^. Foram consideradas as categorias de fisioterapia, psicologia e serviço social, por serem as únicas com registro sistemático nos prontuários eletrônicos em ambos os cenários avaliados. Essas categorias atuam de forma não contínua e sob demanda, permitindo quantificar sua presença nos diferentes modelos assistenciais. A enfermagem, a medicina e a nutrição não foram incluídas por constituírem presença contínua e obrigatória durante a internação, inviabilizando sua utilização como variável comparativa.

As variáveis clínicas foram incluídas por sua relevância prognóstica e por potencial influência na definição do modelo assistencial e na intensidade das intervenções no período final da vida^(14,15,16)^. O recorte temporal dos últimos 30 dias foi adotado, conforme consenso descrito na literatura internacional, para avaliação da qualidade do cuidado em final de vida^(11,12,13)^.

Os dados foram organizados em planilha eletrônica e analisados no *software* R (versão 4.3). As variáveis categóricas foram descritas por meio de frequências absolutas e relativas, enquanto as variáveis numéricas foram expressas por meio de média e desvio padrão ou mediana e intervalo interquartílico, conforme a distribuição dos dados. Para a comparação entre os grupos, foram utilizados os testes qui-quadrado ou exato de Fisher para variáveis categóricas e o teste U de Mann-Whitney para variáveis numéricas, pois não apresentaram distribuição normal segundo o teste de Shapiro-Wilk. Adotou-se o nível de significância de 5%.

### Aspectos Éticos

O estudo foi aprovado pelos Comitês de Ética em Pesquisa da Universidade Federal do Estado do Rio de Janeiro (Parecer n^o^ 6.845.555) e do Instituto Nacional de Câncer (Parecer n^o^ 6.950.714), com dispensa do Termo de Consentimento Livre e Esclarecido por se tratar de estudo retrospectivo.

## RESULTADOS

As características sociodemográficas das 370 mulheres com câncer de mama avançado evidenciaram semelhança entre os grupos, sem diferenças estatisticamente significativas. Observou-se maior proporção de mulheres pardas (46,49%), seguidas de brancas (38,11%) e pretas (15,14%). Quanto à escolaridade, predominou o ensino fundamental incompleto ou analfabetismo (36,22%), seguido do ensino médio (33,51%), enquanto níveis mais elevados de escolaridade foram menos frequentes (10,54%) e com leve predominância no grupo CPE. Em relação ao estado civil, o maior porcentual foi de mulheres solteiras (39,73%), seguidas de casadas ou em união consensual (35,95%). No que tange à religião, houve maior prevalência de mulheres católicas (45,68%), seguida de evangélicas (43,24%).

A distribuição do número de metástases diferiu entre os modelos assistenciais (Tabela 1), com significância estatística (p < 0,001). O grupo QPE apresentou maior frequência de três metástases (32,39%), enquanto o grupo CPE apresentou proporções mais elevadas de quatro (27,32%) e cinco ou mais metástases (40,21%). A mediana de metástases foi de 3 (IIQ 2,75–4,00) entre as pacientes submetidas à quimioterapia paliativa e de 4 (IIQ 3–5) entre as acompanhadas pelos CPE (p < 0,001). Quanto aos sítios metastáticos à distância, observou-se maior ocorrência de metástases no sistema nervoso central entre as pacientes em CPE (39,69% vs. 16,48%).

**Tabela 1:** Distribuição do número de sítios e dos locais de metástases à distância em pacientes com câncer de mama avançado segundo o tipo de cuidado recebido – Rio de Janeiro, RJ, Brasil, 2025.

Variáveis	Total (n = 370^1^)	QPE (n = 176^1^)	CPE (n = 194^1^)	Valor de p^2^
**Número de metástases**				**< 0,001**
1	13 (3,51%)	10 (5,68%)	3 (1,55%)	
2	51 (13,78%)	33 (18,75%)	18 (9,28%)	
3	99 (26,76%)	57 (32,39%)	42 (21,65%)	
4	89 (24,05%)	36 (20,45%)	53 (27,32%)	
≥ 5	117 (31,62%)	39 (22,16%)	78 (40,20%)	
Total de metástase	4 (3–5)	3 (2,75–4,00)	4 (3–5)	**< 0,001**
**Locais de metástases à distância**				
Fígado	173 (46,76%)	84 (47,73%)	89 (45,88%)	0,722
Osso	232 (62,70%)	111 (63,07%)	121 (62,37%)	0,890
Pleural	95 (25,68%)	42 (23,86%)	53 (27,32%)	0,447
Pulmão	211 (57,03%)	105 (59,66%)	106 (54,64%)	0,330
Sistema nervoso central	106 (28,65%)	29 (16,48%)	77 (39,69%)	**< 0,001**
Outros^*^	60 (16,22%)	27 (15,34%)	33 (17,01%)	0,664

Legenda: QPE – quimioterapia paliativa endovenosa; CPE – cuidados paliativos especializados.

*Outros inclui adrenal, bexiga, estômago, mama bilateral, mama contralateral, mediastino, medula óssea, ovário, parasternal, partes moles, pericárdio e peritônio;

^1^Dados expressos como número absoluto (n) e porcentagem;

^2^Análises realizadas por teste qui-quadrado de independência. Houve um caso sem registro do número de metástases.

Na Tabela 2, observa-se que a idade ao óbito diferiu entre os grupos, sendo maior entre as pacientes acompanhadas por CPE (58 anos; IIQ 48–68) em comparação às submetidas à QPE (55 anos; IIQ 45–62) (p = 0,004). O tempo de seguimento institucional foi superior no CPE (53 meses; IIQ 30–102) em relação ao grupo QPE (29 meses; IIQ 9–69) (p < 0,001). Não houve diferença quanto à idade na matrícula (p = 0,373) nem quanto à proposta inicial de quimioterapia (p = 0,312).

**Tabela 2:** Indicadores clínicos e assistenciais das pacientes com câncer de mama avançado segundo o tipo de cuidado recebido* – Rio de Janeiro, RJ, Brasil, 2025.

Variáveis	Total (n = 370^1^)	QPE (n = 176^1^)	CPE (n = 194^1^)	Valor de p^2^
Idade na matrícula^**^	50 (41–59)	50 (41–58)	51 (40–60)	0,373
Idade no óbito^**^	56 (46–66)	55 (45–62)	58 (48–68)	0,004
Tempo na instituição^***^	42 (17–87)	29 (9–69)	53 (30–102)	< 0,001
**Proposta da primeira quimioterapia**				0,312
Adjuvante	42 (11,35%)	17 (9,66%)	25 (12,89%)	
Neoadjuvante	178 (48,11%)	81 (46,02%)	97 (50,00%)	
Paliativo	150 (40,54%)	78 (44,32%)	72 (37,11%)	
**Atendimento multiprofissional**				
Fisioterapia	1 (0–3)	1 (0–3)	1 (0–2)	0,134
Psicologia	0 (0–2)	0 (0–0)	2 (1–4)	< 0,001
Serviço social	1 (0–4)	0 (0–0)	4 (1,25–6)	< 0,001
**Recebeu hemocomponentes**				
**Hemácias**				< 0,001
Sim	38 (10,27%)	32 (18,18%)	6 (3,09%)	
Não	332 (89,73%)	144 (81,82%)	188 (96,91%)	
**Plaquetas**				0,011
Sim	6 (1,62%)	6 (3,41%)	0 (0%)	
Não	364 (98,38%)	170 (96,59%)	194 (100%)	
**Plasma**				0,606
Sim	3 (0,81%)	2 (1,14%)	1 (0,82%)
Não	367 (99,19%)	174 (98,86%)	193 (99,48%)
**Número de atendimentos no SPA**	1 (1–2)	2 (1–3)	1 (1–2)	< 0,001
**Número de dias internada**	8 (4–13)	7 (4–12)	9 (3–14)	0,354
**Internada nas últimas 72 horas**	256 (69,19%)	126 (71,59%)	130 (67,01%)	0,341
**Internada nas últimas 48 horas**	285 (77,03%)	140 (79,55%)	145 (74,74%)	0,273
**Internada nas últimas 24 horas**	331 (89,46%)	172 (97,73%)	159 (81,96%)	< 0,001
**Local do óbito**				< 0,001
Internação hospitalar	331 (89,46%)	172 (97,73%)	159 (81,96%)	
Outro hospital	19 (5,14%)	0 (0,00%)	19 (9,79%)	
A caminho do hospital	5 (1,35%)	1 (0,57%)	4 (2,06%)	
Domicílio	10 (2,70%)	0 (0,0%)	10 (5,15%)	
Casa de apoio	2 (0,54%)	0 (0,00%)	2 (1,03%)	

Legenda: QPE – quimioterapia paliativa endovenosa; CPE – cuidados paliativos especializados; SPA – serviço de pronto atendimento.

*A quantidade total de prontuários analisados pode variar em função de informações ausentes *(missings)*, não correspondendo necessariamente ao total da amostra (QPE: n = 176; CPE: n = 194);

variáveis marcadas com “**” estão expressas em anos

e com “***”, em meses;

^1^Dados expressos como mediana (25%–75%) ou valores categóricos como número absoluto (n) e porcentagem (%), conforme apropriado;

^2^Teste de soma de postos de Wilcoxon, qui-quadrado de independência ou teste exato de Fisher, conforme apropriado.

Em relação ao uso de serviços de emergência, o número de atendimentos no SPA foi maior no grupo QPE (mediana de 2; IIQ 1–3) em comparação ao CPE (mediana de 1; IIQ 1–2) (p < 0,001). O número total de dias de internação nos últimos 30 dias não diferiu entre os grupos (p = 0,354), com medianas de nove dias (IIQ 3–14) no CPE e sete dias (IIQ 4–12) no QPE. Na análise da internação nos últimos dias de vida, observou-se maior proporção de pacientes internadas nas últimas 24 horas no grupo QPE (97,73% vs. 81,96%; p < 0,001), sem diferença nas avaliações de 72 e 48 horas (p > 0,05).

Nos últimos 30 dias de vida, observou-se diferença entre os grupos quanto aos atendimentos multiprofissionais. Pacientes em CPE apresentaram maior número de consultas de psicologia e serviço social em comparação ao QPE (p < 0,001 para ambos), enquanto não houve diferença nos atendimentos de fisioterapia (p = 0,134). Em relação ao uso de hemocomponentes, a transfusão de hemácias foi mais frequente no QPE (18,2%) em comparação ao CPE (3,1%) (p < 0,001), correspondendo a 59 e 12 bolsas transfundidas, respectivamente. A utilização de plaquetas ocorreu exclusivamente no grupo QPE (3,4%; p = 0,011). Para transfusão de plasma, não foi observada diferença estatisticamente significativa entre os grupos (p = 0,606).

A maioria dos óbitos ocorreu nos hospitais do cenário do estudo, com maior frequência entre mulheres submetidas à QPE (97,73% vs. 81,96%; p < 0,001). Entre as pacientes acompanhadas pelos CPE, observaram-se óbitos em outras instituições hospitalares (9,79%), domicílios (5,15%), casa de apoio (1,03%) e durante deslocamento (2,06%). No grupo QPE, não houve óbitos domiciliares ou em casa de apoio; um óbito ocorreu durante o trajeto (0,57%); e três prontuários estavam sem registro do local do óbito.

## DISCUSSÃO

As características sociodemográficas analisadas não exerceram influência significativa sobre o percurso assistencial no fim da vida, pois os grupos apresentaram distribuição semelhante quanto à raça, à escolaridade, ao estado civil e à religião. Diante dessa homogeneidade, as diferenças observadas entre os modelos assistenciais avaliados não parecem estar relacionadas às desigualdades sociais, mas possivelmente à forma como o cuidado especializado foi incorporado ao tratamento oncológico. Esse padrão sugere que critérios clínicos, evolução da doença e práticas institucionais de organização do cuidado podem ter desempenhado papel relevante na configuração das trajetórias assistenciais. A distribuição da proposta inicial de tratamento demonstra que a instituição recebe majoritariamente mulheres em estágios localmente avançados ou metastáticos, com predominância de quimioterapia neoadjuvante ou diretamente paliativa e reduzida indicação adjuvante, padrão compatível com o diagnóstico tardio ainda predominante no Brasil^(17,18)^.

Nesse cenário, o maior tempo de acompanhamento institucional observado entre as pacientes encaminhadas aos CPE (intervalo entre a matrícula na instituição e o óbito) pode indicar que, após um período inicial de controle da doença, ocorreram a progressão e a redução gradual das opções de tratamento modificador, culminando no encaminhamento para os CPE, geralmente quando a irreversibilidade clínica já estava estabelecida. Esse arranjo organizacional sugere que o encaminhamento aos CPE ocorre predominantemente de forma tardia, após o esgotamento das terapias modificadoras da doença, em contraste com as recomendações que orientam a integração precoce e paralela dos cuidados paliativos ao tratamento oncológico ativo em diferentes níveis de atenção, incluindo o manejo inicial pela própria equipe assistente e o encaminhamento para equipes especializadas nos casos de maior complexidade clínica ou sintomática^(5,6)^.

Por outro lado, o seguimento mais curto observado entre as pacientes mantidas exclusivamente em QPE pode refletir a biologia mais agressiva de determinados tumores, característica particularmente descrita em mulheres mais jovens, que apresentam maior frequência de subtipos desfavoráveis e necessidade de tratamento sistêmico mais intenso ao longo da trajetória da doença^(19)^. Evidências provenientes de uma grande coorte prospectiva demonstram que, em cenário de doença avançada, a eficácia das terapias sistêmicas paliativas é dependente da biologia tumoral, com ampla variação na sobrevida mesmo após múltiplas linhas de tratamento^(20)^. Nesse contexto, os achados do presente estudo, que identificaram menor idade no óbito entre aquelas mantidas em quimioterapia paliativa, são compatíveis com esse padrão clínico e sugerem a complexidade do manejo terapêutico em tumores mais agressivos. Entretanto, a persistência de tratamentos antineoplásicos, mesmo diante de irreversibilidade clínica, permanece coerente com o fenômeno da inércia terapêutica, descrito na literatura como fator associado ao atraso na integração dos cuidados paliativos e ao risco de intervenções desproporcionais no fim da vida^(21)^.

Diante desses achados, o conjunto de intervenções observadas no final da vida pode ser interpretado à luz do conceito de obstinação ou futilidade terapêutica, amplamente discutido na literatura. Esse conceito refere-se à manutenção de intervenções biomédicas com baixa probabilidade de benefício proporcional diante da irreversibilidade clínica, frequentemente associada à persistência de tratamentos modificadores da doença, ao uso de procedimentos de suporte com benefício limitado e à ausência de planejamento antecipado do cuidado^(11,12)^. Estudos recentes indicam que a fragmentação assistencial e a integração tardia dos CPE favorecem trajetórias de maior intensidade terapêutica, incluindo quimioterapia próxima ao óbito, hospitalizações repetidas e intervenções de suporte potencialmente desproporcionais^(11,12,13, 22)^.

A gravidade clínica observada no momento da entrada em CPE também reforça essa dinâmica assistencial. Nesse grupo, a maior carga metastática e a elevada frequência de acometimento do sistema nervoso central indicam que os cuidados paliativos passam a assumir o acompanhamento apenas quando a doença já apresenta complicações complexas e potencialmente incapacitantes, limitando o impacto de estratégias preventivas e de planejamento antecipado. A incorporação tardia dos CPE sugere um modelo assistencial predominantemente reativo, centrado na falha terapêutica, e não na integração progressiva e paralela ao tratamento oncológico. Esse padrão está alinhado à literatura internacional, que aponta barreiras estruturais, culturais e organizacionais à integração precoce dos cuidados paliativos, mesmo em contextos nos quais sua oferta é recomendada desde o diagnóstico de doenças ameaçadoras à vida^(4,5,6)^.

Apesar do quadro clínico mais avançado, as pacientes acompanhadas pelos CPE não apresentaram maior consumo de recursos hospitalares. Verificou-se menor uso de serviços emergenciais, menor demanda transfusional e menor frequência de internações no hospital especializado nas últimas 24 horas de vida. Esse achado sugere que a redução da agressividade assistencial no final da vida não decorre apenas da gravidade clínica, mas da forma como o cuidado é organizado, particularmente na antecipação de sintomas e na tomada de decisões. A manutenção da quimioterapia próxima ao óbito, prática amplamente documentada como associada à piora da qualidade de vida e ao aumento da intensidade de intervenções agudas, tem benefício clínico limitado e pode reduzir oportunidades de planejamento terapêutico centrado em conforto^(22,23)^.

Outro aspecto que reforça o padrão de maior intensidade terapêutica entre as pacientes submetidas à quimioterapia paliativa foi o uso de hemocomponentes nas semanas finais de vida. Observou-se maior demanda transfusional nesse grupo, com administração de hemácias e plaquetas até os últimos dias antes do óbito. Embora transfusões possam oferecer benefício sintomático transitório, estudos indicam que tais efeitos são breves e pouco expressivos em pacientes em fase terminal^(24)^. Em estudo retrospectivo envolvendo 179 pacientes com câncer avançado acompanhados em cuidados paliativos, identificou-se que apenas 36% apresentaram benefício clínico, geralmente limitado aos 15 dias após o procedimento, com sobrevida mediana de 41 dias, reforçando o benefício limitado dessa intervenção em indivíduos com mau *status* funcional^(25)^.

Embora não tenha havido diferença estatisticamente significativa no total de dias de internação entre os grupos, a mediana discretamente maior observada entre as pacientes acompanhadas pelos CPE (nove vs. sete dias) deve ser interpretada com cautela, pois a ausência de significância estatística não permite afirmar associação direta entre o modelo assistencial e a duração da hospitalização. Ainda assim, essa diferença pode refletir o perfil clínico mais complexo desse grupo, caracterizado por maior carga metastática e acometimento do sistema nervoso central, frequentemente associadas a sintomas neurológicos e ao rápido declínio funcional, além de reduzidas opções terapêuticas^(26)^. Adicionalmente, múltiplos sítios metastáticos e pior estado geral estão entre os fatores associados à piora da sobrevida em câncer de mama avançado^(27)^. Entretanto, a maior necessidade de cuidado não se traduziu em maior utilização de intervenções agudas, sugerindo o papel modulador dos CPE na prevenção de crises e no manejo antecipatório de sintomas, evitando internações reativas e favorecendo decisões terapêuticas alinhadas aos objetivos do cuidado.

Os mecanismos associados ao menor uso de recursos e à menor agressividade assistencial observados entre pacientes acompanhadas por CPE podem ser compreendidos a partir de três dimensões descritas na literatura sobre integração precoce dos cuidados paliativos. A primeira refere-se ao manejo antecipatório de sintomas e à vigilância clínica contínua, capazes de reduzir descompensações agudas e prevenir crises que frequentemente motivam idas à emergência^(4,5)^. A segunda envolve a tomada de decisão compartilhada e o planejamento antecipado dos cuidados, que permitem alinhar expectativas, evitar intervenções fúteis e reorientar prioridades terapêuticas diante da irreversibilidade clínica^(4,6)^. A terceira relaciona-se à coordenação interdisciplinar do percurso assistencial, integrando dimensões físicas, emocionais, sociais e espirituais^(5,7,28)^.

Neste estudo, as pacientes acompanhadas por CPE receberam maior número de atendimentos de psicologia e serviço social nas últimas semanas de vida, evidenciando a concretização de uma abordagem multiprofissional voltada ao manejo do sofrimento emocional e social, reconhecido como componente essencial da qualidade do cuidado oncológico^(28)^. Esses mecanismos favorecem escolhas terapêuticas mais alinhadas aos valores das pacientes, reduzindo decisões precipitadas diante de crises agudas e modulando o padrão de hospitalização no fim da vida^(4,6)^. Evidências recentes reforçam a relevância desse modelo integrado, demonstrando que a fragmentação assistencial e a negligência das dimensões emocionais, sociais, espirituais, existenciais e relacionais constituem fontes de sofrimento evitável e favorecem padrões reativos de cuidado^(28)^. Superar tais lacunas requer equipes multiprofissionais capazes de oferecer apoio psicológico, social e espiritual ao longo da trajetória da doença, promovendo decisões coerentes com valores, prevenindo intervenções desproporcionais e modulando o uso de recursos no fim da vida^(3)^. Esses elementos dialogam com os achados deste estudo, sugerindo que a presença ativa de equipes de CPE contribui para um cuidado menos agressivo, mais contínuo e alinhado às necessidades e preferências das pacientes.

Adicionalmente, observou-se aumento da proporção de pacientes hospitalizadas à medida que se aproximava o óbito, correspondendo a 69,19% nas últimas 72 horas, 77,03% nas últimas 48 horas e 89,46% nas 24 horas finais. Embora o declínio clínico terminal seja frequentemente acompanhado pela necessidade de internação para manejo de sintomas intensos, como dor, dispneia, agitação e delirium terminal^(4)^, observou-se diferença significativa entre os grupos apenas nas últimas 24 horas de vida, com maior proporção de hospitalização entre pacientes submetidas à quimioterapia paliativa (97,73%), quando comparadas àquelas acompanhadas pelos CPE (81,96%; p < 0,001). Esse padrão pode refletir a manutenção de estratégias terapêuticas modificadoras da doença até fases muito próximas ao óbito, aumentando a dependência de suporte hospitalar e a probabilidade de intervenções intensivas nas horas finais de vida. Em contextos nos quais o planejamento antecipado do cuidado é limitado ou ocorre tardiamente, a persistência dessas estratégias pode favorecer hospitalizações reativas e maior intensidade terapêutica no período final da vida.

Entre as pacientes acompanhadas por CPE, identificou-se maior proporção de óbitos fora do hospital de referência, incluindo outros hospitais (9,79%) e o domicílio (5,15%). Embora tais achados não permitam inferir relação causal direta entre o modelo assistencial e o local de morte, eles sugerem que suporte clínico contínuo, planejamento antecipado e articulação territorial podem reduzir a necessidade de internação em hospital de alta complexidade, redistribuindo os cuidados conforme necessidades clínicas e disponibilidade da rede. No contexto brasileiro, estudo nacional demonstra que, embora grande parte dos pacientes oncológicos manifeste o desejo de morrer em casa, esse desfecho é frequentemente dificultado por insegurança familiar, ausência de suporte especializado e falhas de comunicação no processo assistencial^(29)^.

O modelo de CPE analisado neste estudo caracteriza-se pela articulação entre atendimento ambulatorial, acompanhamento domiciliar e telemonitoramento, integrados à Atenção Primária à Saúde (APS) e a unidades hospitalares de menor complexidade, favorecendo a continuidade do cuidado em locais próximos ao domicílio quando clinicamente apropriado. Nesses contextos, a internação não é necessariamente evitada, mas pode ser realocada de forma mais fluida no território, reduzindo potencialmente a dependência exclusiva do hospital especializado e permitindo o cuidado em unidades mais adequadas à situação clínica e condições familiares. Essa organização dialoga com as diretrizes recentes de organização dos cuidados paliativos no sistema de saúde brasileiro. A Portaria GM/MS n^o^ 3.681/2024 destaca a territorialização e a coordenação intersetorial dos cuidados paliativos, com integração entre serviços especializados, APS e tecnologias de comunicação, visando assegurar a continuidade do cuidado, reduzir os deslocamentos desnecessários e apoiar as decisões compartilhadas sobre o local de morte. Ao ampliar as possibilidades de cuidado fora do hospital de alta complexidade, essa organização assistencial reforça a relevância de redes articuladas e estratégias centradas na pessoa, favorecendo percursos assistenciais compatíveis com necessidades clínicas, preferências e dignidade no processo de morrer^(8)^. Essa perspectiva também se alinha à Agenda 2030, particularmente com o Objetivo de Desenvolvimento Sustentável 3 e sua meta 3.8, que promove a cobertura universal de saúde, na qual os cuidados paliativos são reconhecidos como componente essencial^(9)^.

Nesse contexto, os resultados deste estudo também permitem refletir sobre possíveis implicações para a prática da enfermagem na coordenação e na continuidade do cuidado ao longo da trajetória da doença. Profissionais de enfermagem, por permanecerem em contato mais frequente com pacientes e familiares, desempenham função estratégica na vigilância clínica contínua, na identificação precoce de sintomas e na facilitação da comunicação entre equipe, pacientes e cuidadores. Essa atuação favorece o reconhecimento antecipado de necessidades paliativas, contribui para o planejamento do cuidado e pode apoiar decisões terapêuticas mais alinhadas aos objetivos de cuidado e à condição clínica das pacientes. Evidências recentes apontam que enfermeiros com formação em oncologia e cuidados paliativos podem contribuir para a integração dessas abordagens à prática oncológica, ampliando a capacidade das equipes de saúde de identificar precocemente necessidades paliativas e apoiar o manejo de sintomas e a comunicação com pacientes e familiares ao longo da trajetória da doença^(30)^.

Os resultados deste estudo reforçam que as diferenças observadas entre os grupos parecem decorrer principalmente da forma como o cuidado é organizado ao longo da trajetória da doença, evidenciando a influência do modelo assistencial na intensidade das intervenções no final da vida. Pesquisas internacionais recentes apontam que sistemas oncológicos que articulam de forma estruturada o cuidado paliativo, mesmo quando essa integração ocorre tardiamente, tendem a favorecer maior coerência entre intervenções clínicas, necessidades dos pacientes e objetivos de cuidado, com redução de procedimentos desproporcionais e melhor alinhamento terapêutico^(7)^. À luz desses referenciais, os achados desta investigação contribuem para demonstrar que a presença ativa de equipes de CPE, ainda que introduzida apenas nas fases avançadas da doença, desempenha papel relevante na modulação do uso de recursos, na prevenção de intervenções fúteis e na promoção de um cuidado mais contínuo, seguro e centrado na pessoa no final da vida.

Este estudo apresenta limitações inerentes ao delineamento retrospectivo e à utilização exclusiva de dados secundários, dependentes da qualidade e da completude dos registros eletrônicos. Os grupos comparados não foram constituídos de forma randomizada, o que pode ter introduzido viés de seleção, especialmente porque o encaminhamento aos CPE tende a ocorrer em fases mais avançadas da doença. A análise concentrou-se em uma única instituição pública de referência, o que pode restringir a generalização dos resultados para cenários com menor disponibilidade de insumos, equipes multiprofissionais ou suporte territorial. Não foram avaliadas variáveis psicossociais, preferências das pacientes ou medidas de qualidade percebida do cuidado, que poderiam aprofundar a compreensão da trajetória no fim da vida. Além disso, não foram aplicados modelos multivariados, uma vez que o foco do estudo foi descritivo e comparativo, sem intenção de produzir inferências causais. Apesar dessas limitações, os achados oferecem evidências ainda pouco exploradas no contexto brasileiro ao demonstrar diferenças entre modelos assistenciais no final da vida, contribuindo para o aprimoramento dos CPE e para o planejamento de políticas públicas em oncologia.

## CONCLUSÃO

Os achados deste estudo indicam que o modelo assistencial predominante nos últimos 30 dias de vida esteve associado a diferentes padrões de utilização de serviços de saúde entre mulheres com câncer de mama avançado. Mesmo com perfis sociodemográficos semelhantes, as pacientes acompanhadas pelos CPE apresentaram menor utilização de serviços de emergência, menor uso de hemocomponentes e menor frequência de internação nas últimas 24 horas de vida em hospital especializado, apesar de apresentarem maior carga metastática, incluindo maior comprometimento do sistema nervoso central.

Embora não seja possível estabelecer relações de causalidade, os resultados sugerem que a forma como o cuidado é organizado pode influenciar a trajetória terapêutica no final da vida. Estratégias como suporte multiprofissional, comunicação estruturada, planejamento antecipado do cuidado e articulação entre serviços podem contribuir para maior alinhamento entre as decisões clínicas e os objetivos de cuidado no final da vida. Nesse cenário, é possível refletir sobre as possíveis implicações para a prática da enfermagem na coordenação do cuidado e na navegação assistencial ao longo da trajetória da doença, contribuindo para decisões terapêuticas mais alinhadas às necessidades e prioridades das pacientes no final da vida.

Investigações futuras, especialmente com delineamentos prospectivos, poderão contribuir para analisar como fatores clínicos e características dos modelos assistenciais se relacionam com os desfechos no fim da vida, ampliando a compreensão sobre estratégias que favoreçam um cuidado proporcional, centrado na pessoa e alinhado às necessidades de pacientes com câncer avançado.

## DISPONIBILIDADE DE DADOS

 Todo o conjunto de dados que dá suporte aos resultados deste estudo foi publicado no próprio artigo.

# Translation: Itinerarios asistenciales para mujeres con cáncer de mama avanzado en la fase final de la vida*

**DOI:** 10.1590/1980-220X-REEUSP-2025-0553es

**Authors:** Danielle Copello Vaz, Ana Luisa Teixeira da Costa Durante, Livia Costa de Oliveira, Livia Gomes da Silva, Andrea dos Santos Garcia, Sonia Regina de Souza, Daniel Aragão Machado, Roberto Carlos Lyra da Silva, Suzi Maria Fernandes de Farias, Emanuel Pereira dos Santos, Davi da Silveira Barroso Alves, Carlos Roberto Lyra da Silva

**Affiliations:** 1 Instituto Nacional de Câncer, Hospital do Câncer IV, Ambulatório, Rio de Janeiro, RJ, Brasil.; 2 Instituto Nacional de Câncer, Hospital do Câncer IV, Internação hospitalar, Rio de Janeiro, RJ, Brasil.; 3 Instituto Nacional de Câncer, Hospital do Câncer III, Educação continuada, Rio de Janeiro, RJ, Brasil.; 4 Universidade Federal do Rio de Janeiro, Escola de Enfermagem Anna Nery, Enfermagem Médico-Cirúrgica, Rio de Janeiro, RJ, Brasil.; 5 Universidade Federal do Estado do Rio de Janeiro, Escola de Enfermagem Alfredo Pinto, Enfermagem Médico-Cirúrgica, Rio de Janeiro, RJ, Brasil.; 6 Universidade Federal do Estado do Rio de Janeiro, Escola de Enfermagem Alfredo Pinto, Enfermagem Fundamental, Rio de Janeiro, RJ, Brasil.; 7 Universidade Federal do Estado do Rio de Janeiro, Hospital Universitário Gaffree-Guinle, Anestesiologia, Rio de Janeiro, RJ, Brasil.; 8 Universidade Federal do Estado do Rio de Janeiro, Hospital Universitário Gaffree-Guinle, Pediatria, Rio de Janeiro, RJ, Brasil.; 9 Universidade Federal do Estado do Rio de Janeiro, Centro de Ciências Exatas e Tecnologia, Métodos Quantitativos, Rio de Janeiro, RJ, Brasil.

**Keywords:** Neoplasias de la Mama, Cuidados Paliativos, Atención al Paciente, Indicadores de Servicios, Muerte.

## Abstract

**Objetivo:**

Comparar las trayectorias de atención de mujeres con cáncer de mama avanzado en los últimos 30 días de vida, analizando las diferencias clínicas y la utilización de servicios de salud entre pacientes sometidas a quimioterapia paliativa intravenosa (QPI) y aquellas atendidas por equipos de cuidados paliativos especializados (CPE).

**Método:**

Estudio retrospectivo basado en el análisis de historias clínicas electrónicas de 370 mujeres que fallecieron entre 2019 y 2023, distribuidas en QPI (n = 176) y CPE (n = 194). Las comparaciones se realizaron mediante las pruebas de chi-cuadrado, exacta de Fisher o Mann-Whitney (p < 0,05).

**Resultados:**

Los pacientes en CPE presentaron una mayor carga metastásica (4; RIC 3–5; p < 0,001), mayor afectación del sistema nervioso central (39,69% frente a 16,48%; p < 0,001) y mayor atención multidisciplinaria (p < 0,001). Presentaron menor uso de atención de urgencias (1; RIC 1–2; p < 0,001) y menos hospitalizaciones en las últimas 24 horas de vida (p < 0,001).

**Conclusión:**

El seguimiento por equipos de CPE se asoció con trayectorias de atención menos agresivas al final de la vida, incluso en pacientes con mayor complejidad clínica.

## INTRODUCCIÓN

El cáncer de mama avanzado sigue siendo un desafío global, sobre todo debido a las diferencias estructurales en el acceso al diagnóstico oportuno, a los tratamientos que modifican el curso de la enfermedad y a la continuidad de la atención. Evidencias recientes muestran que, en 2022, el cáncer de mama se mantuvo como el tipo más frecuente entre las mujeres, con aproximadamente 2,3 millones de nuevos casos y cerca de 670 000 muertes, y la relación mortalidad/incidencia varía del 17 % en países altamente desarrollados al 56 % en países de bajos ingresos. Las proyecciones indican que, para 2050, podría producirse un aumento global del 68 % en las muertes por cáncer de mama, superando el 160 % en países de desarrollo medio y bajo, lo que pone de manifiesto disparidades persistentes relacionadas con la infraestructura asistencial, la capacidad diagnóstica y la oferta de terapias^(1)^. En este contexto, tales desigualdades también se reflejan en la oferta global de cuidados paliativos. Una clasificación internacional basada en el marco regulatorio de la Organización Mundial de la Salud puso de manifiesto profundas diferencias entre países y regiones en la implementación de servicios paliativos, especialmente en naciones de ingresos bajos y medios, donde el acceso sigue siendo limitado y heterogéneo^(2)^.

Ante la progresión de la enfermedad, las necesidades clínicas y sociales se vuelven complejas, exigiendo enfoques orientados a la calidad de vida, al control de los síntomas y a la toma de decisiones compartida. Desde esta perspectiva, los cuidados paliativos constituyen un enfoque multidimensional orientado al alivio del sufrimiento derivado de afecciones que amenazan la vida, integrando dimensiones físicas, psicosociales, espirituales y existenciales, de manera continua y articulada, a lo largo del curso de la enfermedad^(3)^. Las directrices internacionales destacan que la integración temprana de los cuidados paliativos debe ocurrir desde el diagnóstico de enfermedades que amenazan la vida, de manera paralela al tratamiento modificador de la enfermedad, siendo complementaria y no sustitutiva de las terapias activas, incluso cuando hay indicación de terapia sistémica^(4,5 6)^. Este principio sustenta la incorporación sistemática de los cuidados paliativos al tratamiento oncológico, especialmente en el contexto de la enfermedad avanzada, favoreciendo un enfoque integrado, proporcional al pronóstico y centrado en la calidad de vida^(5,7)^. En este contexto, los cuidados paliativos especializados (CPE) están indicados principalmente en situaciones de mayor complejidad clínica, como síntomas refractarios o sufrimiento de difícil control^(5,6)^.

En Brasil, la Política Nacional de Cuidados Paliativos avanzó con la publicación de la Orden Ministerial GM/MS n.^o^ 3.681/2024, que establece directrices y organiza los servicios en niveles asistenciales, con monitoreo de la calidad de la atención en el Sistema Único de Salud^(8)^. Al reconocer los cuidados paliativos como un elemento estructurante de la Red de Atención de Salud, la norma amplía la visibilidad de los cuidados al final de la vida y establece las bases para la planificación y la articulación entre los niveles de atención, además de permitir la evaluación de la atención a las personas con enfermedades que ponen en riesgo la vida. Estas orientaciones están en consonancia con los compromisos éticos y sociales de la Agenda 2030, que se centra en la equidad, el derecho universal a la salud, la dignidad humana y la reducción de las desigualdades —dimensiones que impactan en la atención a las personas con enfermedades avanzadas^(9)^.

A pesar de los avances consolidados en la literatura internacional, especialmente en lo que se refiere a la integración temprana de los cuidados paliativos y a la reducción de la agresividad asistencial al final de la vida^(5,7)^, aún se necesitan análisis comparativos que investiguen cómo los diferentes modelos asistenciales se asocian con los patrones de atención en el período final de la vida de las mujeres con cáncer de mama avanzado, especialmente en el contexto brasileño. Los estudios indican que la derivación a equipos de cuidados paliativos sigue siendo predominantemente tardía, lo que restringe las oportunidades de planificación anticipada y de un enfoque centrado en el confort^(10)^. Paralelamente, las investigaciones evidencian un elevado uso de servicios hospitalarios e intervenciones intensivas en los últimos meses de vida de los pacientes oncológicos, lo que refuerza la persistencia de patrones asistenciales potencialmente agresivos al final de la vida^(11)^. En este escenario, resulta relevante comparar cómo la persistencia del tratamiento oncológico activo y el seguimiento por parte de un equipo especializado en cuidados paliativos se relacionan con la configuración de las trayectorias al final de la vida.

A efectos conceptuales, se distingue entre itinerario asistencial y modelo asistencial. El itinerario asistencial se refiere al recorrido concreto vivido por la paciente en el sistema de salud, incluyendo los servicios a los que se accede, las transiciones entre niveles de atención y las intervenciones realizadas a lo largo de la enfermedad y el final de la vida. El modelo asistencial, por su parte, corresponde a la lógica organizativa predominante de la atención que orienta las decisiones clínicas e institucionales. En el contexto de este estudio, el modelo asistencial se operacionalizó como: (1) CPE, caracterizado por el seguimiento formal por parte de un equipo especializado que prioriza las medidas de confort y el control sintomático; o (2) persistencia del tratamiento oncológico activo con quimioterapia paliativa intravenosa (QPI), caracterizada por el mantenimiento de intervenciones modificadoras de la enfermedad en el período final de la vida.

Así, este estudio se justifica por la necesidad de analizar cómo los diferentes modelos asistenciales se asocian a la configuración de la atención de las mujeres con cáncer de mama avanzado al final de la vida, contribuyendo a la identificación de patrones asistenciales y ofreciendo información para la planificación y la organización de la atención. Se parte de la hipótesis de que las mujeres atendidas por CPE presentan una menor frecuencia de intervenciones consideradas agresivas al final de la vida en comparación con aquellas que continúan con un tratamiento oncológico activo.

Ante lo expuesto, este estudio tuvo como objetivo comparar las trayectorias asistenciales de las mujeres con cáncer de mama avanzado en los últimos 30 días de vida, analizando las diferencias clínicas y de uso de los servicios de salud entre las pacientes sometidas a QPI y aquellas atendidas por equipos de CPE.

## MÉTODO

### Tipo de Estudio

Se trata de un estudio retrospectivo, cuantitativo, observacional y analítico, basado en datos secundarios extraídos de historias clínicas electrónicas. Se compararon dos grupos de mujeres con cáncer de mama avanzado que fallecieron entre 2019 y 2023, según el modelo de atención predominante en los últimos 30 días de vida.

### Lugar del Estudio

La investigación se llevó a cabo en un hospital público de alta complejidad, referente nacional en oncología, ubicado en el estado de Río de Janeiro. La institución cuenta con un centro dedicado al tratamiento del cáncer de mama y una unidad exclusiva de CPE, lo que permite la comparación entre diferentes trayectorias asistenciales.

### Población y Criterios de Selección

Se incluyeron mujeres con diagnóstico confirmado de cáncer de mama avanzado que fallecieron en la institución entre enero de 2019 y diciembre de 2023. Se excluyeron los casos con un segundo tumor primario, inconsistencia diagnóstica (Clasificación Internacional de Enfermedades inadecuada o error de registro) o COVID-19 en los últimos 30 días. La muestra correspondió a la totalidad de los casos elegibles en el período analizado (n = 370).

El proceso de selección implicó la identificación de los fallecimientos en el sistema institucional, seguida de la aplicación de los criterios de elegibilidad y la clasificación de las pacientes según el modelo asistencial predominante en los 30 días previos al fallecimiento. La comparación analizó diferentes trayectorias de atención en el período final de la vida, considerando la organización asistencial como eje estructurante del análisis. Para ello, las participantes se agruparon según el modelo asistencial predominante en los últimos 30 días de vida.

El grupo QPI estuvo compuesto por mujeres que recibieron QPI en los últimos 30 días de vida, atendidas en servicios oncológicos sin integración en un equipo de CPE. En este modelo, la atención se mantuvo estructurada bajo la lógica del tratamiento oncológico activo, con un enfoque predominante en el mantenimiento de terapias antineoplásicas y en el manejo reactivo de las complicaciones clínicas.

El grupo CPE incluyó a mujeres previamente sometidas a QPI a lo largo de su trayectoria asistencial y que, en los últimos 30 días de vida, se encontraban bajo seguimiento exclusivo de un equipo de CPE, sin recibir terapias modificadoras de la enfermedad en ese período. El modelo de atención en este grupo se centró en el control y la anticipación de los síntomas, en la toma de decisiones compartida y en la coordinación interdisciplinaria de la atención.

La clasificación consideró exclusivamente el modelo de atención predominante en los últimos 30 días de vida, independientemente del momento de la transición a la CPE. Todas las pacientes incluidas recibieron QPI en algún momento de la trayectoria asistencial. Las cohortes se distinguieron en cuanto a la continuidad o interrupción de los cuidados paliativos en el período final de la vida.

En la institución estudiada, la incorporación a los cuidados paliativos ocurre tras la suspensión de las terapias modificadoras de la enfermedad, lo que caracteriza una transición secuencial entre el tratamiento oncológico activo y los cuidados paliativos. Así, las pacientes no son sometidas simultáneamente a los dos modelos de atención. Además, el seguimiento exclusivo por parte de la CPE no incluye medidas invasivas de soporte vital avanzado, como el ingreso en la Unidad de Terapia Intensiva, la intubación orotraqueal o la reanimación cardiopulmonar. Por esta razón, dichas variables no se incluyeron en el análisis comparativo entre los grupos. Esta característica organizacional debe tenerse en cuenta en la interpretación de los hallazgos, ya que puede influir en el perfil clínico de las pacientes en el momento de la transición y en las intervenciones realizadas en el período final de vida.

### Recolección y Análisis de Datos

La recolección de datos se realizó mediante un análisis retrospectivo de los expedientes médicos electrónicos institucionales, del sistema de gestión hospitalaria y de bases de datos administrativas complementarias. La extracción de datos fue realizada por la investigadora principal, utilizando la misma ficha estandarizada de Recolección de datos para ambos grupos, elaborada específicamente para el estudio con base en la literatura sobre indicadores de intensidad terapéutica y agresividad al final de la vida en oncología^(11,12,13)^. El instrumento fue previamente probado en una muestra piloto para verificar la claridad y la integridad de los campos. La ficha incluyó variables sociodemográficas, clínicas y asistenciales referidas a los últimos 30 días de vida.

Se analizaron variables sociodemográficas (edad al ingreso y al fallecimiento, raza, estado civil, nivel educativo y religión), variables clínicas (carga metastásica, número y localización de las metástasis a distancia, tiempo de seguimiento institucional y propuesta inicial de quimioterapia) e indicadores asistenciales relacionados con el final de la vida. El tiempo de seguimiento institucional corresponde al intervalo entre el ingreso institucional de la paciente y el fallecimiento.

Los indicadores asistenciales incluyeron la administración de quimioterapia cerca del fallecimiento, el número de visitas al servicio de urgencias (SPA), el número de días de hospitalización, el lugar del fallecimiento y el derivado tardío a los CPE, variables descritas en la literatura como marcadores de intensidad terapéutica y potencial agresividad de la atención al final de la vida en pacientes oncológicos^(11,12,13)^.

Además, se analizaron el uso de hemocomponentes y la atención multiprofesional en los últimos 30 días de vida, ya que reflejan la intensidad de la atención hospitalaria y el patrón organizativo de los cuidados en el período final, lo que permite comprender las trayectorias asistenciales entre los diferentes modelos. El análisis de la atención multiprofesional se basó en la literatura que reconoce el enfoque interdisciplinario como componente central de la calidad de los cuidados paliativos y de la organización asistencial al final de la vida^(4,14)^. Se consideraron las categorías de fisioterapia, psicología y servicio social, por ser las únicas con registro sistemático en los expedientes médicos electrónicos en ambos escenarios evaluados. Estas categorías actúan de manera no continua y bajo demanda, lo que permite cuantificar su presencia en los diferentes modelos asistenciales. La enfermería, la medicina y la nutrición no se incluyeron por constituir una presencia continua y obligatoria durante la hospitalización, lo que inviabiliza su utilización como variable comparativa.

Se incluyeron las variables clínicas por su relevancia pronóstica y por su influencia potencial en la definición del modelo de atención y en la intensidad de las intervenciones en el período final de la vida^(14,15,16)^. Se adoptó el intervalo temporal de los últimos 30 días, de acuerdo con el consenso descrito en la literatura internacional, para la evaluación de la calidad de la atención al final de la vida^(11,12,13)^.

Los datos se organizaron en una hoja de cálculo y se analizaron con el software R (versión 4.3). Las variables categóricas se describieron mediante frecuencias absolutas y relativas, mientras que las variables numéricas se expresaron mediante la media y la desviación estándar o la mediana y el intervalo intercuartílico, según la distribución de los datos. Para la comparación entre los grupos, se utilizaron las pruebas de Chi-cuadrado o exacta de Fisher para las variables categóricas y la prueba U de Mann-Whitney para las variables numéricas, ya que no presentaban una distribución normal según la prueba de Shapiro-Wilk. Se adoptó un nivel de significación del 5 %.

### Aspectos éticos

El estudio fue aprobado por los Comités de Ética en Investigación de la Universidad Federal del Estado de Río de Janeiro (Dictamen n.^o^ 6.845.555) y del Instituto Nacional del Cáncer (Dictamen n.^o^ 6.950.714), con exención del Término de Consentimiento Libre e Informado al tratarse de un estudio retrospectivo.

## RESULTADOS

Las características sociodemográficas de las 370 mujeres con cáncer de mama avanzado mostraron similitudes entre los grupos, sin diferencias estadísticamente significativas. Se observó una mayor proporción de mujeres de raza mestiza (46,49 %), seguidas de las de raza blanca (38,11 %) y las de raza negra (15,14 %). En cuanto a la escolaridad, predominó la educación primaria incompleta o el analfabetismo (36,22 %), seguido de la educación secundaria (33,51 %), mientras que los niveles más altos de escolaridad fueron menos frecuentes (10,54 %) y con un ligero predominio en el grupo CPE.

En cuanto al estado civil, el mayor porcentaje correspondió a mujeres solteras (39,73 %), seguidas de casadas o en unión libre (35,95 %). En cuanto a la religión, hubo una mayor prevalencia de mujeres católicas (45,68 %), seguidas de evangélicas (43,24 %).

La distribución del número de metástasis difirió entre los modelos de atención (Tabla 1), con significación estadística (p < 0,001). El grupo QPI presentó una mayor frecuencia de tres metástasis (32,39 %), mientras que el grupo CPE presentó proporciones más elevadas de cuatro (27,32 %) y cinco o más metástasis (40,21 %). La mediana de metástasis fue de 3 (Intervalo Intercuartílico – IIC 2,75–4,00) entre las pacientes sometidas a quimioterapia paliativa y de 4 (IIC 3–5) entre las atendidas por el CPE (p < 0,001). En cuanto a los sitios metastásicos a distancia, se observó una mayor incidencia de metástasis en el sistema nervioso central entre las pacientes en CPE (39,69 % frente a 16,48 %).

**Tabla 1:** Distribución del número de focos y de los sitios de metástasis a distancia en pacientes con cáncer de mama avanzado según el tipo de atención recibida – Río de Janeiro, RJ, Brasil, 2025.

Variables	Total (n = 370^1^)	QPI (n = 176^1^)	CPE (n = 194^1^)	Valor de p^2^
**Número de metástasis**				**< 0,001**
1	13 (3,51%)	10 (5,68%)	3 (1,55%)
2	51 (13,78%)	33 (18,75%)	18 (9,28%)
3	99 (26,76%)	57 (32,39%)	42 (21,65%)
4	89 (24,05%)	36 (20,45%)	53 (27,32%)
≥ 5	117 (31,62%)	39 (22,16%)	78 (40,20%)
Total de metástasis	4 (3–5)	3 (2,75–4,00)	4 (3–5)	**< 0,001**
**Localizaciones de metástasis a distancia**
Hígado	173 (46,76%)	84 (47,73%)	89 (45,88%)	0,722
Hueso	232 (62,70%)	111 (63,07%)	121 (62,37%)	0,890
Pleural	95 (25,68%)	42 (23,86%)	53 (27,32%)	0,447
Pulmón	211 (57,03%)	105 (59,66%)	106 (54,64%)	0,330
Sistema nervioso central	106 (28,65%)	29 (16,48%)	77 (39,69%)	< 0,001
Otros^*^	60 (16,22%)	27 (15,34%)	33 (17,01%)	0,664

Leyenda: QPI: quimioterapia paliativa intravenosa; CPE: cuidados paliativos especializados.

*Otros incluye suprarrenal, vejiga, estómago, mama bilateral, mama contralateral, mediastino, médula ósea, ovario, paraesternal, partes blandas, pericardio y peritoneo;

^1^Datos expresados como número absoluto (n) y porcentaje;

^2^Análisis realizados mediante la prueba de chi-cuadrado de independencia. Hubo un caso sin registro del número de metástasis.

En la Tabla 2 se observa que la edad al fallecimiento difirió entre los grupos, siendo mayor entre las pacientes seguidas mediante CPE (58 años; IQ 48–68) en comparación con las sometidas a QPI (55 años; IQ 45–62) (p = 0,004). El tiempo de seguimiento institucional fue mayor en el CPE (53 meses; IQ 30–102) en comparación con el grupo QPI (29 meses; IQ 9–69) (p < 0,001). No hubo diferencias en cuanto a la edad en el momento de la inscripción (p = 0,373) ni en cuanto a la propuesta inicial de quimioterapia (p = 0,312).

**Tabla 2:** Indicadores clínicos y asistenciales de las pacientes con cáncer de mama avanzado según el tipo de atención recibida* – Río de Janeiro, RJ, Brasil, 2025.

Variables	Total (n = 370^1^)	QPI (n = 176^1^)	CPE (n = 194^1^)	Valor de p^2^
Edad al matricularse^**^	50 (41–59)	50 (41–58)	51 (40–60)	0,373
Edad al fallecer^**^	56 (46–66)	55 (45–62)	58 (48–68)	0,004
Tiempo en la institución^***^	42 (17–87)	29 (9–69)	53 (30–102)	< 0,001
**Propuesta de la primera quimioterapia**				0,312
Adyuvante	42 (11,35%)	17 (9,66%)	25 (12,89%)	
Neoadyuvante	178 (48,11%)	81 (46,02%)	97 (50,00%)	
Paliativo	150 (40,54%)	78 (44,32%)	72 (37,11%)	
**Atención multiprofesional**
Fisioterapia	1 (0–3)	1 (0–3)	1 (0–2)	0,134
Psicología	0 (0–2)	0 (0–0)	2 (1–4)	< 0,001
Trabajo social	1 (0–4)	0 (0–0)	4 (1,25–6)	< 0,001
**Recibió hemoderivados**				
**Glóbulos rojos**				< 0,001
Sí	38 (10,27%)	32 (18,18%)	6 (3,09%)
No	332 (89,73%)	144 (81,82%)	188 (96,91%)
**Plaquetas**				0,011
Sí	6 (1,62%)	6 (3,41%)	0 (0%)	
No	364 (98,38%)	170 (96,59%)	194 (100%)	
**Plasma**				0,606
Sí	3 (0,81%)	2 (1,14%)	1 (0,82%)	
No	367 (99,19%)	174 (98,86%)	193 (99,48%)	
**Número de consultas en el SPA**	1 (1–2)	2 (1–3)	1 (1–2)	< 0,001
**Número de días de hospitalización**	8 (4–13)	7 (4–12)	9 (3–14)	0,354
**Ingresada en las últimas 72 horas**	256 (69,19%)	126 (71,59%)	130 (67,01%)	0,341
**Ingresada en las últimas 48 horas**	285 (77,03%)	140 (79,55%)	145 (74,74%)	0,273
**Ingresada en las últimas 24 horas**	331 (89,46%)	172 (97,73%)	159 (81,96%)	< 0,001
**Lugar del fallecimiento**				< 0,001
Ingreso hospitalario	331 (89,46%)	172 (97,73%)	159 (81,96%)	
Otro hospital	19 (5,14%)	0 (0,00%)	19 (9,79%)	
De camino al hospital	5 (1,35%)	1 (0,57%)	4 (2,06%)	
Domicilio	10 (2,70%)	0 (0,0%)	10 (5,15%)	
Centro de acogida	2 (0,54%)	0 (0,00%)	2 (1,03%)	

Leyenda: QPI: quimioterapia paliativa intravenosa; CPE: cuidados paliativos especializados; SPA: servicio de urgencias.

*El número total de historias clínicas analizadas puede variar debido a datos faltantes (missings), por lo que no se corresponde necesariamente con el total de la muestra (QPI: n = 176; CPE: n = 194);

las variables marcadas con «**» se expresan en años

y las marcadas con «***», en meses;

^1^Datos expresados como mediana (25 %–75 %) o valores categóricos como número absoluto (n) y porcentaje (%), según corresponda;

^2^Prueba de suma de rangos de Wilcoxon, chi-cuadrado de independencia o prueba exacta de Fisher, según corresponda.

En cuanto al uso de los servicios de urgencias, el número de visitas al SPA fue mayor en el grupo QPI (mediana de 2; IIC 1–3) en comparación con el CPE (mediana de 1; IIC 1–2) (p < 0,001). El número total de días de hospitalización en los últimos 30 días no difirió entre los grupos (p = 0,354), con medianas de nueve días (IIC 3–14) en el CPE y siete días (IIC 4–12) en el QPI. En el análisis de la hospitalización en los últimos días de vida, se observó una mayor proporción de pacientes hospitalizadas en las últimas 24 horas en el grupo QPI (97,73 % frente a 81,96 %; p < 0,001), sin diferencias en las evaluaciones de 72 y 48 horas (p > 0,05).

En los últimos 30 días de vida, se observó una diferencia entre los grupos en cuanto a la atención multiprofesional. Los pacientes en CPE presentaron un mayor número de consultas de psicología y servicio social en comparación con el QPI (p < 0,001 para ambos), mientras que no hubo diferencias en la atención de fisioterapia (p = 0,134). En cuanto al uso de hemocomponentes, la transfusión de glóbulos rojos fue más frecuente en el QPI (18,2 %) en comparación con el CPE (3,1 %) (p < 0,001), lo que corresponde a 59 y 12 bolsas transfundidas, respectivamente. El uso de plaquetas se produjo exclusivamente en el grupo QPI (3,4 %; p = 0,011). En cuanto a la transfusión de plasma, no se observó una diferencia estadísticamente significativa entre los grupos (p = 0,606).

La mayoría de las muertes se produjeron en los hospitales del escenario del estudio, con mayor frecuencia entre las mujeres sometidas a QPI (97,73 % frente a 81,96 %; p < 0,001). Entre las pacientes atendidas por los CPE, se observaron fallecimientos en otros centros hospitalarios (9,79 %), domicilios (5,15 %), centros de acogida (1,03 %) y durante el traslado (2,06 %). En el grupo de QPI, no se produjeron fallecimientos en el domicilio ni en centros de acogida; se produjo un fallecimiento durante el traslado (0,57 %); y en tres historias clínicas no constaba el lugar del fallecimiento.

## DISCUSIÓN

Las características sociodemográficas analizadas no ejercieron una influencia significativa sobre el proceso de atención al final de la vida, ya que los grupos presentaron una distribución similar en cuanto a raza, nivel educativo, estado civil y religión. Ante esta homogeneidad, las diferencias observadas entre los modelos de atención evaluados no parecen estar relacionadas con las desigualdades sociales, sino posiblemente con la forma en que la atención especializada se incorporó al tratamiento oncológico. Este patrón sugiere que los criterios clínicos, la evolución de la enfermedad y las prácticas institucionales de organización de la atención pueden haber desempeñado un papel relevante en la configuración de las trayectorias asistenciales. La distribución de la propuesta inicial de tratamiento demuestra que la institución atiende mayoritariamente a mujeres en estadios localmente avanzados o metastásicos, con predominio de quimioterapia neoadyuvante o directamente paliativa y una indicación adyuvante reducida, patrón compatible con el diagnóstico tardío que aún predomina en Brasil^(17,18)^.

En este escenario, el mayor tiempo de seguimiento institucional observado entre las pacientes derivadas a los CPE (intervalo entre la inscripción en la institución y el fallecimiento) puede indicar que, tras un período inicial de control de la enfermedad, se produjo la progresión y la reducción gradual de las opciones de tratamiento modificador, lo que culminó en la derivación a los CPE, generalmente cuando la irreversibilidad clínica ya estaba establecida. Este arreglo organizativo sugiere que la derivación a los CPE se produce predominantemente de forma tardía, tras el agotamiento de las terapias modificadoras de la enfermedad, en contraste con las recomendaciones que orientan hacia la integración temprana y paralela de los cuidados paliativos al tratamiento oncológico activo en diferentes niveles de atención, incluyendo el manejo inicial por parte del propio equipo asistencial y el derivado a equipos especializados en los casos de mayor complejidad clínica o sintomática^(5,6)^.

Por otro lado, el seguimiento más corto observado entre las pacientes mantenidas exclusivamente en QPI puede reflejar la biología más agresiva de determinados tumores, característica descrita particularmente en mujeres más jóvenes, que presentan una mayor frecuencia de subtipos desfavorables y la necesidad de un tratamiento sistémico más intenso a lo largo de la trayectoria de la enfermedad^(19)^. Las evidencias procedentes de una gran cohorte prospectiva demuestran que, en el escenario de la enfermedad avanzada, la eficacia de las terapias sistémicas paliativas depende de la biología tumoral, con una amplia variación en la supervivencia incluso tras múltiples líneas de tratamiento^(20)^. En este contexto, los hallazgos del presente estudio, que identificaron una menor edad al momento del fallecimiento entre las pacientes que continuaron con quimioterapia paliativa, son compatibles con este patrón clínico y sugieren la complejidad del manejo terapéutico en tumores más agresivos. Sin embargo, la persistencia de los tratamientos antineoplásicos, incluso ante la irreversibilidad clínica, sigue siendo coherente con el fenómeno de la inercia terapéutica, descrito en la literatura como un factor asociado al retraso en la integración de los cuidados paliativos y al riesgo de intervenciones desproporcionadas al final de la vida^(21)^.

Ante estos hallazgos, el conjunto de intervenciones observadas al final de la vida puede interpretarse a la luz del concepto de obstinación o futilidad terapéutica, ampliamente discutido en la literatura. Este concepto se refiere al mantenimiento de intervenciones biomédicas con baja probabilidad de beneficio proporcional ante la irreversibilidad clínica, frecuentemente asociado a la persistencia de tratamientos modificadores de la enfermedad, al uso de procedimientos de soporte con beneficio limitado y a la ausencia de planificación anticipada de la atención^(11–12)^. Estudios recientes indican que la fragmentación de la atención y la integración tardía de los CPE favorecen trayectorias de mayor intensidad terapéutica, incluyendo quimioterapia cercana al fallecimiento, hospitalizaciones repetidas e intervenciones de soporte potencialmente desproporcionadas^(11,12,13, 22)^.

La gravedad clínica observada en el momento del ingreso en cuidados paliativos también refuerza esta dinámica asistencial. En este grupo, la mayor carga metastásica y la elevada frecuencia de afectación del sistema nervioso central indican que los cuidados paliativos asumen el seguimiento solo cuando la enfermedad ya presenta complicaciones complejas y potencialmente incapacitantes, lo que limita el impacto de las estrategias preventivas y de planificación anticipada. La incorporación tardía de los CPE sugiere un modelo de atención predominantemente reactivo, centrado en el fracaso terapéutico, y no en la integración progresiva y paralela al tratamiento oncológico. Este patrón concuerda con la literatura internacional, que señala barreras estructurales, culturales y organizacionales para la integración temprana de los cuidados paliativos, incluso en contextos en los que se recomienda su oferta desde el diagnóstico de enfermedades que amenazan la vida^(4,5,6)^.

A pesar del cuadro clínico más avanzado, las pacientes atendidas por los CPE no presentaron un mayor consumo de recursos hospitalarios. Se observó un menor uso de servicios de emergencia, una menor demanda de transfusiones y una menor frecuencia de ingresos en el hospital especializado en las últimas 24 horas de vida. Este hallazgo sugiere que la reducción de la agresividad asistencial al final de la vida no se debe únicamente a la gravedad clínica, sino a la forma en que se organiza la atención, particularmente en la anticipación de los síntomas y en la toma de decisiones. El mantenimiento de la quimioterapia cerca del momento del fallecimiento, práctica ampliamente documentada como asociada al empeoramiento de la calidad de vida y al aumento de la intensidad de las intervenciones agudas, tiene un beneficio clínico limitado y puede reducir las oportunidades de planificación terapéutica centrada en el confort^(22,23)^.

Otro aspecto que refuerza el patrón de mayor intensidad terapéutica entre las pacientes sometidas a quimioterapia paliativa fue el uso de hemocomponentes en las últimas semanas de vida. Se observó una mayor demanda transfusional en este grupo, con administración de glóbulos rojos y plaquetas hasta los últimos días antes del fallecimiento. Aunque las transfusiones pueden ofrecer un beneficio sintomático transitorio, los estudios indican que dichos efectos son breves y poco significativos en pacientes en fase terminal^(24)^.

En un estudio retrospectivo que incluyó a 179 pacientes con cáncer avanzado atendidos en cuidados paliativos, se identificó que solo el 36 % presentó un beneficio clínico, generalmente limitado a los 15 días posteriores al procedimiento, con una sobrevida mediana de 41 días, lo que refuerza el beneficio limitado de esta intervención en individuos con mal estado funcional^(25)^.

Aunque no hubo una diferencia estadísticamente significativa en el total de días de hospitalización entre los grupos, la mediana ligeramente mayor observada entre las pacientes atendidas por los CPE (nueve frente a siete días) debe interpretarse con cautela, ya que la ausencia de significación estadística no permite afirmar una asociación directa entre el modelo de atención y la duración de la hospitalización. Aun así, esta diferencia puede reflejar el perfil clínico más complejo de este grupo, caracterizado por una mayor carga metastásica y afectación del sistema nervioso central, frecuentemente asociadas a síntomas neurológicos y al rápido deterioro funcional, además de opciones terapéuticas reducidas^(26)^. Además, las metástasis en múltiples sitios y un peor estado general se encuentran entre los factores asociados con una peor supervivencia en el cáncer de mama avanzado^(27)^. Sin embargo, la mayor necesidad de cuidados no se tradujo en un mayor uso de intervenciones agudas, lo que sugiere el papel modulador de los CPE en la prevención de crisis y en el manejo anticipatorio de los síntomas, evitando hospitalizaciones reactivas y favoreciendo decisiones terapéuticas alineadas con los objetivos de la atención.

Los mecanismos asociados al menor uso de recursos y a la menor agresividad asistencial observados entre las pacientes atendidas por CPE pueden entenderse a partir de tres dimensiones descritas en la literatura sobre la integración temprana de los cuidados paliativos. La primera se refiere al manejo anticipatorio de los síntomas y a la vigilancia clínica continua, capaces de reducir las descompensaciones agudas y prevenir las crisis que con frecuencia motivan visitas a la sala de emergencias^(4,5)^. La segunda implica la toma de decisiones compartida y la planificación anticipada de los cuidados, lo que permite alinear las expectativas, evitar intervenciones inútiles y reorientar las prioridades terapéuticas ante la irreversibilidad clínica^(4,6)^. La tercera se relaciona con la coordinación interdisciplinaria del proceso de atención, integrando las dimensiones físicas, emocionales, sociales y espirituales^(5,7,28)^.

En este estudio, las pacientes atendidas por el CPE recibieron un mayor número de consultas de psicología y servicio social en las últimas semanas de vida, lo que evidencia la concreción de un enfoque multiprofesional orientado al manejo del sufrimiento emocional y social, reconocido como un componente esencial de la calidad de la atención oncológica^(28)^.

Estos mecanismos favorecen elecciones terapéuticas más alineadas con los valores de las pacientes, reduciendo las decisiones precipitadas ante crisis agudas y modulando el patrón de hospitalización al final de la vida^(4,6)^. Evidencias recientes refuerzan la relevancia de este modelo integrado, demostrando que la fragmentación asistencial y la negligencia de las dimensiones emocionales, sociales, espirituales, existenciales y relacionales constituyen fuentes de sufrimiento evitable y favorecen patrones reactivos de atención^(28)^. Superar estas deficiencias requiere equipos multiprofesionales capaces de ofrecer apoyo psicológico, social y espiritual a lo largo del curso de la enfermedad, promoviendo decisiones coherentes con los valores, previniendo intervenciones desproporcionadas y modulando el uso de recursos al final de la vida^(3)^. Estos elementos concuerdan con los hallazgos de este estudio, lo que sugiere que la presencia activa de equipos de CPE contribuye a una atención menos agresiva, más continua y alineada con las necesidades y preferencias de las pacientes.

Además, se observó un aumento en la proporción de pacientes hospitalizadas a medida que se acercaba el fallecimiento, correspondiendo al 69,19 % en las últimas 72 horas, al 77,03 % en las últimas 48 horas y al 89,46 % en las últimas 24 horas. Aunque el deterioro clínico terminal suele ir acompañado de la necesidad de hospitalización para el manejo de síntomas intensos, como dolor, disnea, agitación y delirio terminal^(4)^, solo se observó una diferencia significativa entre los grupos en las últimas 24 horas de vida, con una mayor proporción de hospitalización entre las pacientes sometidas a quimioterapia paliativa (97,73 %), en comparación con aquellas atendidas por los CPE (81,96 %; p < 0,001). Este patrón puede reflejar el mantenimiento de estrategias terapéuticas modificadoras de la enfermedad hasta fases muy cercanas al fallecimiento, lo que aumenta la dependencia del soporte hospitalario y la probabilidad de intervenciones intensivas en las últimas horas de vida. En contextos en los que la planificación anticipada de la atención es limitada o se produce tardíamente, la persistencia de estas estrategias puede favorecer hospitalizaciones reactivas y una mayor intensidad terapéutica en el período final de la vida.

Entre las pacientes atendidas por el CPE, se identificó una mayor proporción de fallecimientos fuera del hospital de referencia, incluyendo otros hospitales (9,79 %) y el domicilio (5,15 %). Aunque estos hallazgos no permiten inferir una relación causal directa entre el modelo de atención y el lugar de fallecimiento, sugieren que el apoyo clínico continuo, la planificación anticipada y la coordinación territorial pueden reducir la necesidad de hospitalización en un hospital de alta complejidad, redistribuyendo la atención según las necesidades clínicas y la disponibilidad de la red. En el contexto brasileño, un estudio nacional demuestra que, aunque gran parte de los pacientes oncológicos manifiestan el deseo de morir en casa, este desenlace se ve a menudo dificultado por la inseguridad familiar, la falta de apoyo especializado y las fallas de comunicación en el proceso asistencial^(29)^.

El modelo de CPE analizado en este estudio se caracteriza por la articulación entre la atención ambulatoria, el seguimiento domiciliario y el telemonitoreo, integrados a la Atención Primaria de Salud (APS) y a unidades hospitalarias de menor complejidad, lo que favorece la continuidad de la atención en lugares cercanos al domicilio cuando sea clínicamente apropiado. En estos contextos, la hospitalización no se evita necesariamente, pero puede reubicarse de manera más fluida en el territorio, reduciendo potencialmente la dependencia exclusiva del hospital especializado y permitiendo la atención en unidades más adecuadas a la situación clínica y las condiciones familiares. Esta organización está en consonancia con las recientes directrices de organización de los cuidados paliativos en el sistema de salud brasileño. La Orden GM/MS n.^o^ 3.681/2024 destaca la territorialización y la coordinación intersectorial de los cuidados paliativos, con la integración entre servicios especializados, APS y tecnologías de comunicación, con el objetivo de asegurar la continuidad de la atención, reducir los desplazamientos innecesarios y apoyar las decisiones compartidas sobre el lugar de la muerte. Al ampliar las posibilidades de atención fuera de los hospitales de alta complejidad, esta organización asistencial refuerza la relevancia de las redes articuladas y las estrategias centradas en la persona, favoreciendo trayectorias asistenciales compatibles con las necesidades clínicas, las preferencias y la dignidad en el proceso de morir^(8)^. Esta perspectiva también se alinea con la Agenda 2030, particularmente con el Objetivo de Desarrollo Sostenible 3 y su meta 3.8, que promueve la cobertura universal de salud, en la que los cuidados paliativos se reconocen como un componente esencial^(9)^.

En este contexto, los resultados de este estudio también permiten reflexionar sobre posibles implicaciones para la práctica de la enfermería en la coordinación y la continuidad de la atención a lo largo de la trayectoria de la enfermedad. Los profesionales de enfermería, al estar en contacto más frecuente con los pacientes y sus familiares, desempeñan una función estratégica en la vigilancia clínica continua, en la identificación temprana de síntomas y en la facilitación de la comunicación entre el equipo, los pacientes y los cuidadores. Esta actuación favorece el reconocimiento anticipado de las necesidades paliativas, contribuye a la planificación de la atención y puede respaldar decisiones terapéuticas más alineadas con los objetivos de la atención y la condición clínica de las pacientes. Evidencias recientes indican que los enfermeros con formación en oncología y cuidados paliativos pueden contribuir a la integración de estos enfoques en la práctica oncológica, ampliando la capacidad de los equipos de salud para identificar precocemente las necesidades paliativas y apoyar el manejo de los síntomas y la comunicación con pacientes y familiares a lo largo del curso de la enfermedad^(30)^.

Los resultados de este estudio refuerzan la idea de que las diferencias observadas entre los grupos parecen derivarse principalmente de la forma en que se organiza la atención a lo largo del curso de la enfermedad, lo que pone de manifiesto la influencia del modelo asistencial en la intensidad de las intervenciones al final de la vida.

Investigaciones internacionales recientes señalan que los sistemas oncológicos que articulan de manera estructurada los cuidados paliativos, incluso cuando esta integración se produce tardíamente, tienden a favorecer una mayor coherencia entre las intervenciones clínicas, las necesidades de los pacientes y los objetivos de la atención, con una reducción de los procedimientos desproporcionados y una mejor alineación terapéutica^(7)^. A la luz de estos referentes, los hallazgos de esta investigación contribuyen a demostrar que la presencia activa de equipos de cuidados paliativos, aunque se introduzca solo en las fases avanzadas de la enfermedad, desempeña un papel relevante en la modulación del uso de recursos, en la prevención de intervenciones inútiles y en la promoción de una atención más continua, segura y centrada en la persona al final de la vida.

Este estudio presenta limitaciones inherentes al diseño retrospectivo y al uso exclusivo de datos secundarios, que dependen de la calidad y la exhaustividad de los registros electrónicos. Los grupos comparados no se constituyeron de manera aleatoria, lo que puede haber introducido un sesgo de selección, especialmente porque la derivación a los CPE tiende a ocurrir en fases más avanzadas de la enfermedad. El análisis se centró en una única institución pública de referencia, lo que puede limitar la generalización de los resultados a entornos con menor disponibilidad de insumos, equipos multiprofesionales o apoyo territorial. No se evaluaron variables psicosociales, preferencias de las pacientes ni medidas de la calidad percibida de la atención, que podrían profundizar la comprensión de la trayectoria al final de la vida. Además, no se aplicaron modelos multivariados, ya que el enfoque del estudio fue descriptivo y comparativo, sin intención de producir inferencias causales. A pesar de estas limitaciones, los hallazgos ofrecen evidencia aún poco explorada en el contexto brasileño al demostrar diferencias entre los modelos de atención al final de la vida, lo que contribuye al mejoramiento de los CPE y a la planificación de políticas públicas en oncología.

## CONCLUSIÓN

Los hallazgos de este estudio indican que el modelo de atención predominante en los últimos 30 días de vida se asoció con diferentes patrones de uso de los servicios de salud entre las mujeres con cáncer de mama avanzado. A pesar de tener perfiles sociodemográficos similares, las pacientes atendidas por los CPE presentaron un menor uso de servicios de emergencia, un menor uso de hemocomponentes y una menor frecuencia de hospitalización en las últimas 24 horas de vida en un hospital especializado, a pesar de presentar una mayor carga metastásica, incluyendo un mayor compromiso del sistema nervioso central.

Aunque no es posible establecer relaciones de causalidad, los resultados sugieren que la forma en que se organiza la atención puede influir en la trayectoria terapéutica al final de la vida. Estrategias como el apoyo multiprofesional, la comunicación estructurada, la planificación anticipada de la atención y la articulación entre servicios pueden contribuir a una mayor alineación entre las decisiones clínicas y los objetivos de la atención al final de la vida. En este contexto, es posible reflexionar sobre las posibles implicaciones para la práctica de la enfermería en la coordinación de la atención y la navegación asistencial a lo largo de la trayectoria de la enfermedad, contribuyendo a que las decisiones terapéuticas estén más alineadas con las necesidades y prioridades de las pacientes al final de la vida.

Las investigaciones futuras, especialmente con diseños prospectivos, podrán contribuir a analizar cómo los factores clínicos y las características de los modelos asistenciales se relacionan con los resultados al final de la vida, ampliando la comprensión sobre estrategias que favorezcan una atención proporcional, centrada en la persona y alineada con las necesidades de los pacientes con cáncer avanzado.

## DISPONIBILIDAD DE DATOS

Todo el conjunto de datos que respalda los resultados de este estudio se publicó en el propio artículo.
